# Disruption of the Intestinal Mucosal Barrier Induced by High Fructose and Restraint Stress Is Regulated by the Intestinal Microbiota and Microbiota Metabolites

**DOI:** 10.1128/spectrum.04698-22

**Published:** 2023-01-31

**Authors:** Jiayu Yu, Tianlong Liu, Qingyun Guo, Zixu Wang, Yaoxing Chen, Yulan Dong

**Affiliations:** a Key Laboratory of Precision Nutrition and Food Quality, Ministry of Education, College of Veterinary Medicine, China Agricultural University, Beijing, People’s Republic of China; b Milu Conservation Research Unit, Beijing Milu Ecological Research Center, Beijing, People’s Republic of China; Jilin University

**Keywords:** intestinal microbiota, intestinal mucosal barrier, microbiota metabolites

## Abstract

Environmental (restraint stress) and dietary (high fructose) factors are key triggers for flares of inflammatory bowel disease; however, the mechanisms involved in this phenomenon are not fully elucidated. This study aimed to investigate the mechanisms by which restraint stress and high fructose damage the intestinal mucosal immune barrier. The feces of C57BL/6J mice were subjected to 16S rRNA and untargeted metabolome sequencing, and the intestinal histological structure was analyzed by immunohistochemistry and immunofluorescence staining. The mRNA and protein levels of the intestinal protein were analyzed by reverse transcription-PCR (RT-PCR), Western blotting, and enzyme-linked immunosorbent assay (ELISA). The metabolites of the microbiota were tested *in vitro*, and Akkermansia muciniphila was used for colonization *in vivo*. Dietary fructose exacerbated the development of restraint stress, with an extensive change in the composition of the gut microbiota and microbial metabolites. The disturbance of the microbiota composition led to an increase in the abundance of histamine and a decrease in the abundance of taurine, which inhibited the expression of tight junction and MUC2 proteins, destroyed the function of NLRP6, and reduced intestinal autophagy level; this in turn disrupted the function of colonic goblet cells to secrete mucus, leading to defects in the intestinal mucosal barrier, which ultimately codrives colon autoinflammation. However, *A. muciniphila* supplementation counteracted damage to the intestinal mucosal barrier by high fructose and restraint stress. Therefore, the gut microbiota and microbiota metabolites play an important role in maintaining microenvironment homeostasis of the intestinal mucosal barrier.

**IMPORTANCE** A high-fructose diet aggravated restraint stress-induced changes in the composition of the intestinal microbiome, in which the abundance of *A. muciniphila* was significantly increased. The high-fructose diet exacerbated restraint stress-induced the changes in the composition of the microbial metabolites, with taurine abundance being downregulated and histamine abundance upregulated. High fructose and restraint stress induced colonic mucosal immune barrier damage, possibly due to changes in the abundance of the microbial metabolites taurine and histamine. Colonization with *A. muciniphila* stimulated the expression of the NLRP6 inflammasome and activated autophagy in goblet cells, thereby producing more new mucins, which could protect the intestinal mucosal barrier.

## INTRODUCTION

Psychosocial stress alters the interaction between the brain and the intestines and may exacerbate a series of diseases, including inflammatory bowel disease (IBD) (1) and irritable bowel syndrome (IBS) (2). Stress through physical restraint or exposure to social stressors has been shown to cause changes in the gut microbiome and increase susceptibility to intestinal infections (3). Disturbed gut microbiotas could account for leaky gut and inflammation (4), since disturbed microbiotas can produce metabolites that interact with the host’s intestinal epithelial cells to reduce epithelial barrier proteins, leading to disruption of intercellular junctions and thus reduced intestinal permeability (5). At present, the pathogenesis of these two intestinal diseases is extremely confusing. Studies have found that diet has a dramatic impact on the composition of the gut microbiome and can lead to the spread of pathogens (6). It is well known that some unhealthy dietary regimens are associated with increased incidence of chronic diseases, including hypertensive cardiovascular disease, type 2 diabetes, obesity, and cancer (7).

Fructose is a highly lipogenic sugar that is abundant in processed foods and beverages all over the world. The main dietary sources of fructose are high-fructose corn syrup and sucrose, because both sugars are commonly used to increase the sweetness of beverages and processed foods (8). Sugar-sweetened beverages (SSBs) are the single largest contributor to fructose intake in the United States diet (9). Fructose leads to downregulation of tight junction proteins (TJPs) through a yet-unknown mechanism, leading to deterioration of the gut epithelial barrier (10, 11).

The intestinal barrier mainly consists of the intestinal symbiotic microbiota, mucins, antimicrobial peptides, intestinal epithelial cells, and mucosal lymphatic tissue. In the colon, the mucus layer forms a powerful barrier against the invasion of pathogens and commensal bacteria (12). Symbiotic microorganisms are critical to the integrity of the intestinal epithelial barrier (13). One study found that the mucus layers of germfree mice were much thinner than those of conventionally raised mice, suggesting that the microbiome may contribute to mucus production (14). Of note, the presence of the NOD-like receptor family pyrin domain-containing 6 (NLRP6) inflammasome, an intracellular innate immune sensor, could maintain intestinal microenvironment homeostasis (15). Nlrp6^−/−^ mice were found to have an abnormal expansion of commensal bacterial species in the family *Prevotellaceae* (16). Moreover, these mice exhibited increased susceptibility to colitis, a phenotype that could be transmitted to wild-type mice through cohousing (16). Autophagy is also crucial for the maintenance of intestinal homeostasis. Autophagy defects in intestinal epithelial cells (IEC) have been reported to lead to bacterial translocation and increased inflammation following acute Salmonella infection (17, 18). Another study found that the absence of Atg7, a key autophagy protein in intestinal epithelial cells, significantly altered the composition of the intestinal microbiota and reduced alpha diversity (19).

Akkermansia muciniphila is a Gram-negative, strictly anaerobic bacterium belonging to the phylum *Verrucomicrobia*. Recently, *A. muciniphila* was identified as a mucus-degrading bacterium residing in the mucus layer (20). Although the beneficial role of *A. muciniphila* in metabolic disease (21) has been recognized, its role in intestinal disease has been controversial. *A. muciniphila* exacerbates Salmonella- and *Citrobacter*-induced intestinal inflammation by disrupting host mucus homeostasis (22, 23). The question of whether the increase in mucolytic bacteria is the root cause of the increase in the pathogenesis of colitis has not previously been answered.

In this study, we found that dietary fructose exacerbated the development of restraint stress, an extensive change in the composition of the gut microbiota and microbial metabolites, dysfunction of the intestinal NLRP6 inflammasome, and loss of autophagy levels, with significantly elevated levels of inflammation, leading to the destruction of the intestinal mucosal immune barrier. Taurine, a microbiota metabolite, increased the expression of tight junction and MUC2 proteins and enhanced NLRP6 and autophagy protein expression *in vitro*, while histamine inhibited the expression of NLRP6 protein. In addition, *A. muciniphila* supplementation counteracted damage to the intestinal mucosal barrier caused by high fructose and restraint stress. In conclusion, we identified the gut microbiota and microbiota metabolites as key factors in maintaining intestinal mucosal barrier microenvironment homeostasis.

## RESULTS

### High fructose intake exacerbated disruption of the colonic mucosal immune barrier and induced inflammation under restraint stress conditions.

**FIG 1:**
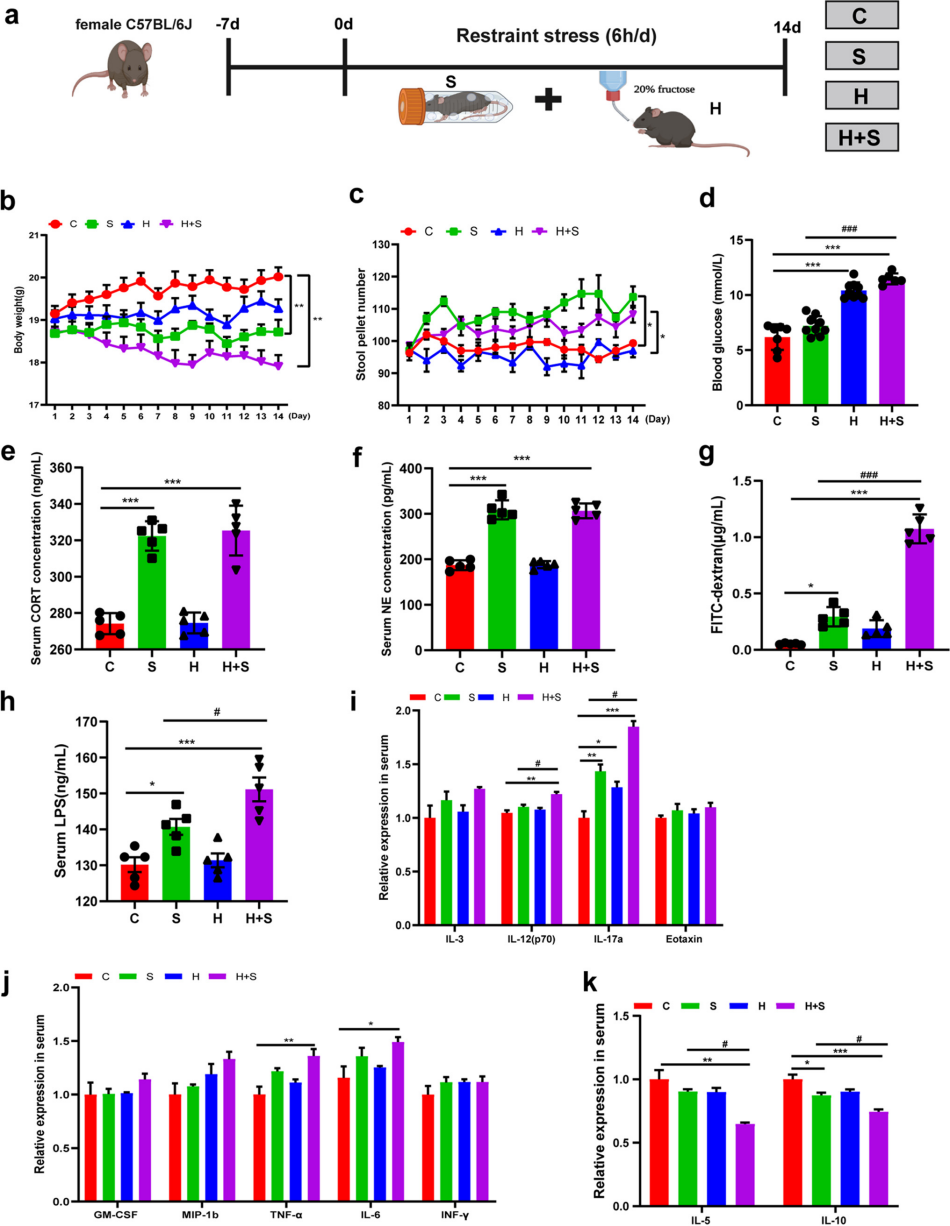
Changes in body weight, intestinal permeability, serum hormones, and inflammatory factors in mice. (a) Study design of experiment I. (b) Daily body weight. (c) Daily stool pellet numbers for five mice over 2 h. (d) Blood glucose levels. (e and f) Secretion of stress hormones CORT (e) and NE (f). (g) Analysis of intestinal permeability using FITC-dextran in serum. Mice received FITC-dextran by gavage. Serum samples were collected 4 h later (*n* = 5). (h) Serum endotoxin levels determined by ELISA. LPS, lipopolysaccharide. (i to k) Cytokines in serum detected by Luminex liquid suspension chip technology. Data are means and standard errors of the means (SEM). *, *P* < 0.05; **, *P* < 0.01; ***, *P* < 0.001 compared with the control group (C). #, *P* < 0.05; ###, *P* < 0.001 compared with the restraint stress group (S). H, high fructose; H+S, high fructose and restraint stress.

Restraint stress for 6 h/day for 14 consecutive days (Fig. 1a) induced weight reduction (*P* < 0.001) and softer stools, consistent with increased stool pellet counts (*P* = 0.011), in five mice within 2 h compared with the control group (Fig. 1b and c). Blood glucose concentration was elevated significantly in the group receiving high fructose and undergoing restraint stress (H+S group) (*P* < 0.001) compared to the control (C) group (Fig. 1d). Both serum corticosterone (CORT) and norepinephrine (NE) are markers of stress, and they increased during stress responses (24). The levels of CORT and NE in serum increased significantly after restraint stress treatment (Fig. 1e and f). At the same time, intestinal permeability increased significantly in the restraint stress (S) (*P* = 0.013) and H+S groups (*P* < 0.001) (Fig. 1g). Serum endotoxin levels also changed significantly in the S (*P* = 0.034) and H+S (*P* < 0.001) groups compared to the control group (Fig. 1h), which was consistent with changes in intestinal permeability. Similarly, the weight of the liver was significantly increased in the high-fructose (H) group (*P* = 0.046) and H+S group (*P* = 0.002) (see Fig. S1A in the supplemental material). The weight of the spleen was significantly increased in the S (*P* = 0.000) and H+S (*P* < 0.001) groups (Fig. S1B). Changes in cytokines in serum were detected by Luminex liquid suspension chip technology. In the H+S group, the expression levels of the proinflammatory cytokines interleukin 12 (IL-12) (p70) (*P* = 0.002), IL-17a (*P* < 0.001), tumor necrosis factor alpha (TNF-α) (*P* = 0.006), and IL-6 (*P* = 0.042) were significantly higher than in the control group; also, the expression levels of IL-12 (p70) (*P* = 0.016) and IL-17a (*P* = 0.004) were significantly higher than in the S group (Fig. 1i and j). The changes in the anti-inflammatory cytokines IL-5 (*P* = 0.002) and IL-10 (*P* = 0.000) were significantly lower in the H+S group than the control group (Fig. 1k).

**FIG 2:**
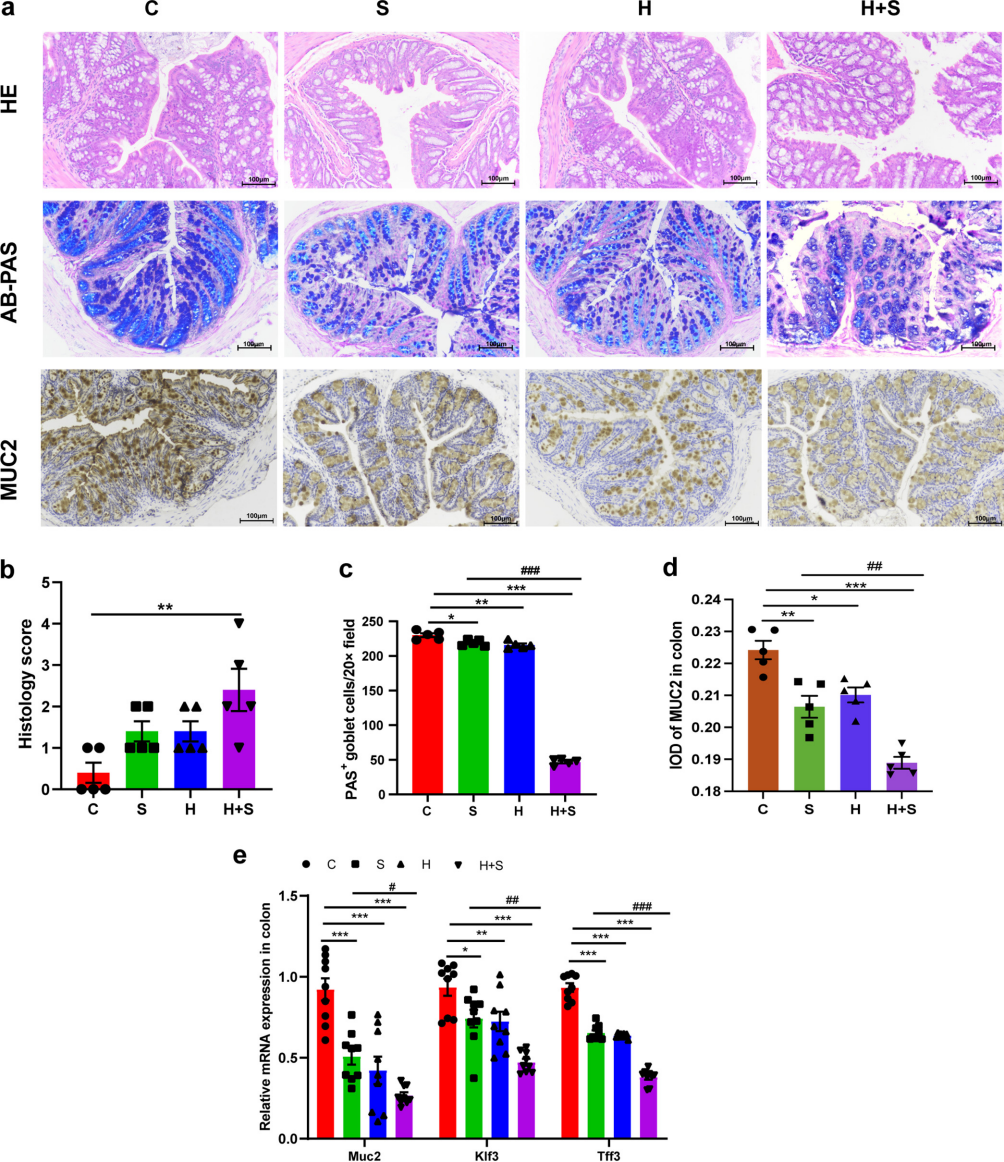
Restraint stress and high-fructose feeding resulted in the destruction of colonic mucosal barrier in mice. (a) Colon histopathology (H&E), AB/PAS-stained colon sections showing goblet cells, and immunohistochemistry staining of MUC2 in intestinal tissue sections Bars, 100 μm (H&E and AB/PAS staining) and 50 μm (MUC2 staining). (b) Histologic scores. (c) Enumeration of goblet cells. (d) IOD of MUC2 in the intestinal tissue from each treatment group (*n* = 5; 20 microscopic images were obtained for each treatment group). (e) Expression of Muc2, Kfl3, and Tff3 was measured by real-time qPCR in mice (*n* = 5 per group). Data are means and SEM. *, *P* < 0.05; **, *P* < 0.01; ***, *P* < 0.001 compared with the control group (C). #, *P* < 0.05; ##, *P* < 0.01; ###, *P* < 0.001 compared with the restraint stress group (S).

**FIG 3:**
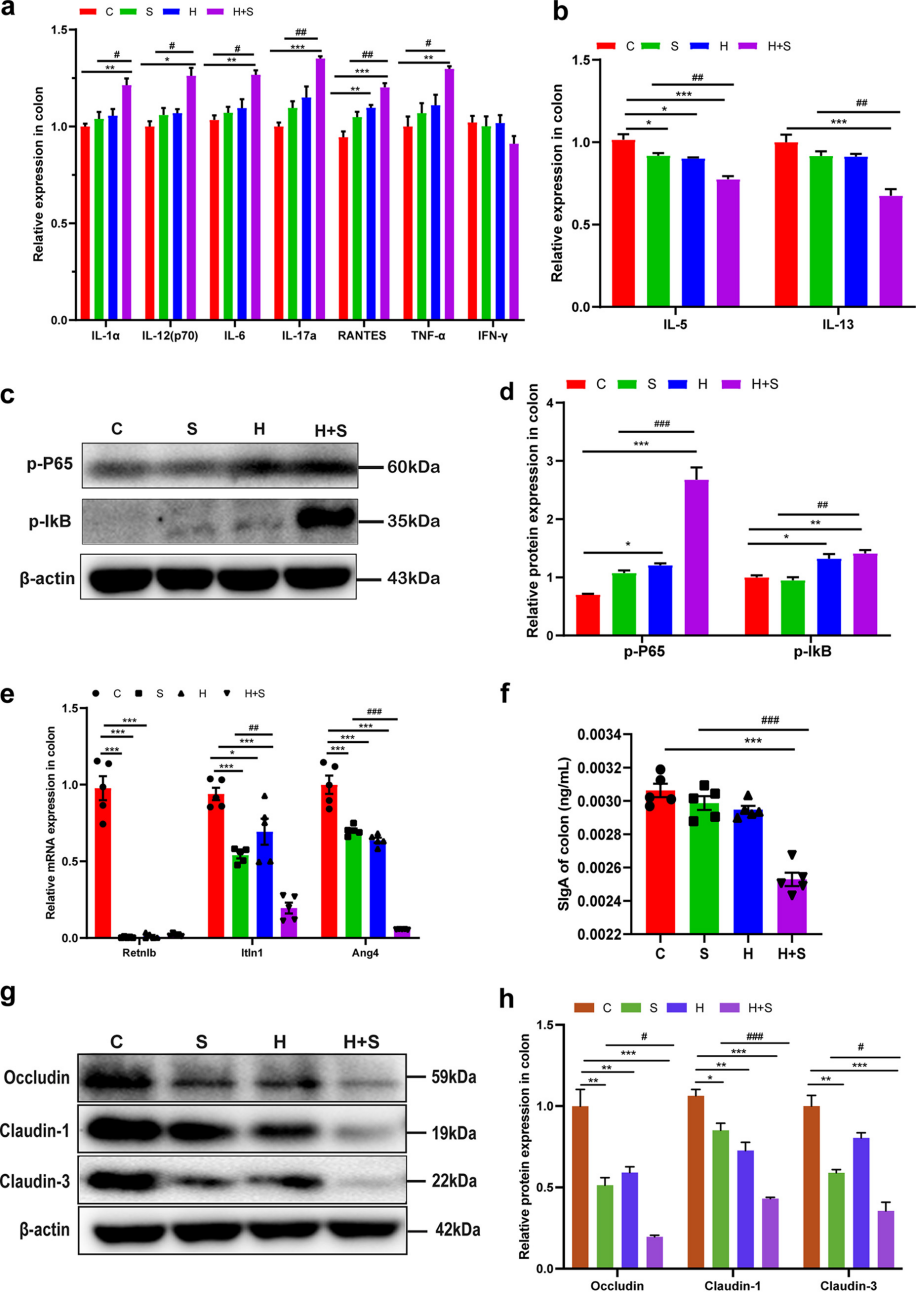
Restraint stress and high-fructose feeding increased colonic inflammation and decreased tight junction protein expression. (a and b) Cytokines in the colon were detected by Luminex liquid suspension chip technology. (c and d) The activation of NF-KB signaling pathway (p-P65 and p-IkB) in the colon was measured by Western blotting (c), and relative protein levels were normalized to β-actin (*n* = 5) (d). (e) Changes in mRNA levels of antimicrobial peptides. (f) SIgA secretion in colon tissue detected by ELISA. (g and h) Expression of colon tight junction protein occludin, claudin-1, claudin-3, and β-actin was examined by Western blotting (g), and relative protein levels were normalized to β-actin (*n* = 5) (h). Data are means and SEM. *, *P* < 0.05; **, *P* < 0.01; ***, *P* < 0.001 compared with the control group (C). #, *P* < 0.05; ##, *P* < 0.01; ###, *P* < 0.001 compared with the restraint stress group (S). H, high fructose; H+S, high fructose and restraint stress.

Next, we examined the effects of high fructose and restraint stress on the intestinal mucosal immune barrier. The histological score showed that the colon tissue of the H+S group had obvious colon tissue damage compared with the control group (*P* = 0.003), manifested as severe villous epithelial atrophy and crypt epithelial loss (Fig. 2a and b). Alcian blue–periodic acid-Schiff (AB-PAS) staining showed that goblet cells were blue-purple and scattered among epithelial cells, mainly in the lower part of the villi. In our research, the number of goblet cells in the H+S group was significantly lower than in the S group (*P* < 0.001) (Fig. 2a and c). MUC2 is secreted primarily by goblet cells and enters the intestinal lumen to form a mucus layer. The integrated optical density (IOD) of MUC2-positive cells in the H+S group was significantly lower than in the S group (*P* = 0.001) (Fig. 2a and d). Tff3 is a goblet cell protein that promotes mucosal repair and protection, and the transcription factor Klf3 participates in barrier function. It was consistent with our prediction that the mRNA levels of Muc2 (*P* = 0.044), Klf3 (*P* = 0.007), and Tff3 (*P* < 0.001) were decreased more severely in the H+S group than the S group (Fig. 2e). We further tested the effect of fructose intake and restraint stress on the antioxidant capacity of the intestine by examining five antioxidant parameters, including antioxidant enzymes (glutathione peroxidase [GSH-Px], superoxide dismutase [SOD], and catalase [CAT]), total antioxidant capability (T-AOC), and malondialdehyde (MDA). Consistent with our prediction, the levels of CAT (*P* < 0.001), GSH-Px (*P* = 0.000), SOD (*P* = 0.002), and CAT (*P* = 0.001) decreased significantly in the H+S group (Fig. S2A to D). However, the level of MDA, the final product of lipid peroxidation, was significantly increased in the H+S (*P* = 0.001) group, compared with the control group (Fig. S2E). The above results indicated that high-fructose treatment could aggravate the stress-induced weakening of colon antioxidants. The levels of the proinflammatory cytokines IL-1α (*P* = 0.017), IL-12 (p70) (*P* = 0.015), IL-6 (*P* = 0.011), IL-17a (*P* = 0.039), RANTES (*P* = 0.008), and TNF-α (*P* = 0.030) increased significantly in the H+S group compared with the S group (Fig. 3a). Expression of the anti-inflammatory cytokines IL-5 (*P* = 0.005) and IL-13 (*P* = 0.005) decreased significantly in the H+S group compared with the S group (Fig. 3b). Moreover, high fructose and restraint stress activated the colonic NF-κB pathway. The protein levels of p-P65 (*P* < 0.001) and p-IκB (*P* = 0.002) were increased in the H+S group compared with the S group (Fig. 3c and d). This indicated that the elevated level of intestinal oxidative stress may induce intestinal inflammation.

Through analysis of the reverse transcription-PCR (RT-PCR) results for different antimicrobial peptides, we found that the expression levels of Itln1 (*P* = 0.001) and Ang4 (*P* < 0.001) were significantly lower in the H+S group than the S group (Fig. 3e). Secretory immunoglobulin A (SIgA) is the first line of defense of the intestinal mucosal barrier; the secretion level of SIgA in the H+S group (*P* < 0.001) was significantly lower than in the S group (Fig. 3f). When the expression of tight junction proteins in colon tissue was assessed by Western blotting, the expression levels of occludin (*P* = 0.022), claudin-1 (*P* = 0.000), and claudin-3 (*P* = 0.029) in the H+S group were significantly lower than in the S group (Fig. 3g and h). This indicates that high fructose intake could worsen restraint stress stimulation, aggravate the destruction of intestinal mucosa, reduce the secretion of antimicrobial peptides, and promote the expression of proinflammatory cytokines.

### High fructose intake increased colonic apoptosis and reduced autophagy under restraint stress conditions.

**FIG 4:**
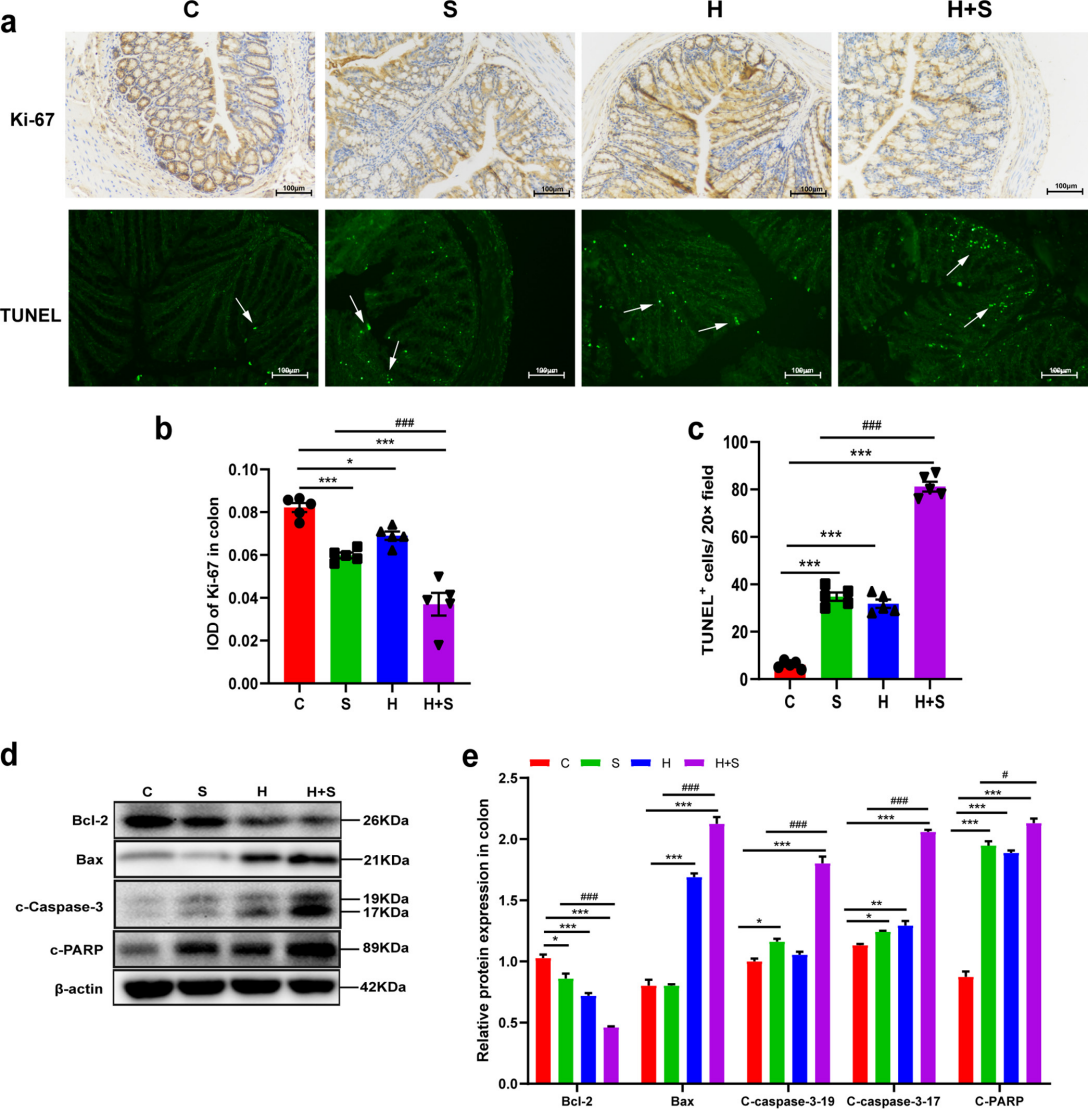
Restraint stress and high-fructose feeding led to intestinal apoptosis. (a) Immunohistochemical staining of Ki-67 in colon sections (bar, 100 μm) and (a) apoptosis measured by TUNEL staining. Arrows show TUNEL-positive cells (green). (b) IOD of Ki-67-positive cells in the colons (*n* = 5). (c) The number of TUNEL-positive cells per 20 magnification was determined (*n* = 5 mice per group). (d and e) Production of the antiapoptotic protein Bcl-2, apoptotic protein Bax, caspase-3, and PARP was examined by Western blotting (d), and relative protein levels were normalized to β-actin (*n* = 5) (e). Data are means and SEM. *, *P* < 0.05; **, *P* < 0.01; ***, *P* < 0.001 compared with the control group (C). #, *P* < 0.05; ###, *P* < 0.001 compared with the restraint stress group (S). H, high fructose; H+S, high fructose and restraint stress.

**FIG 5:**
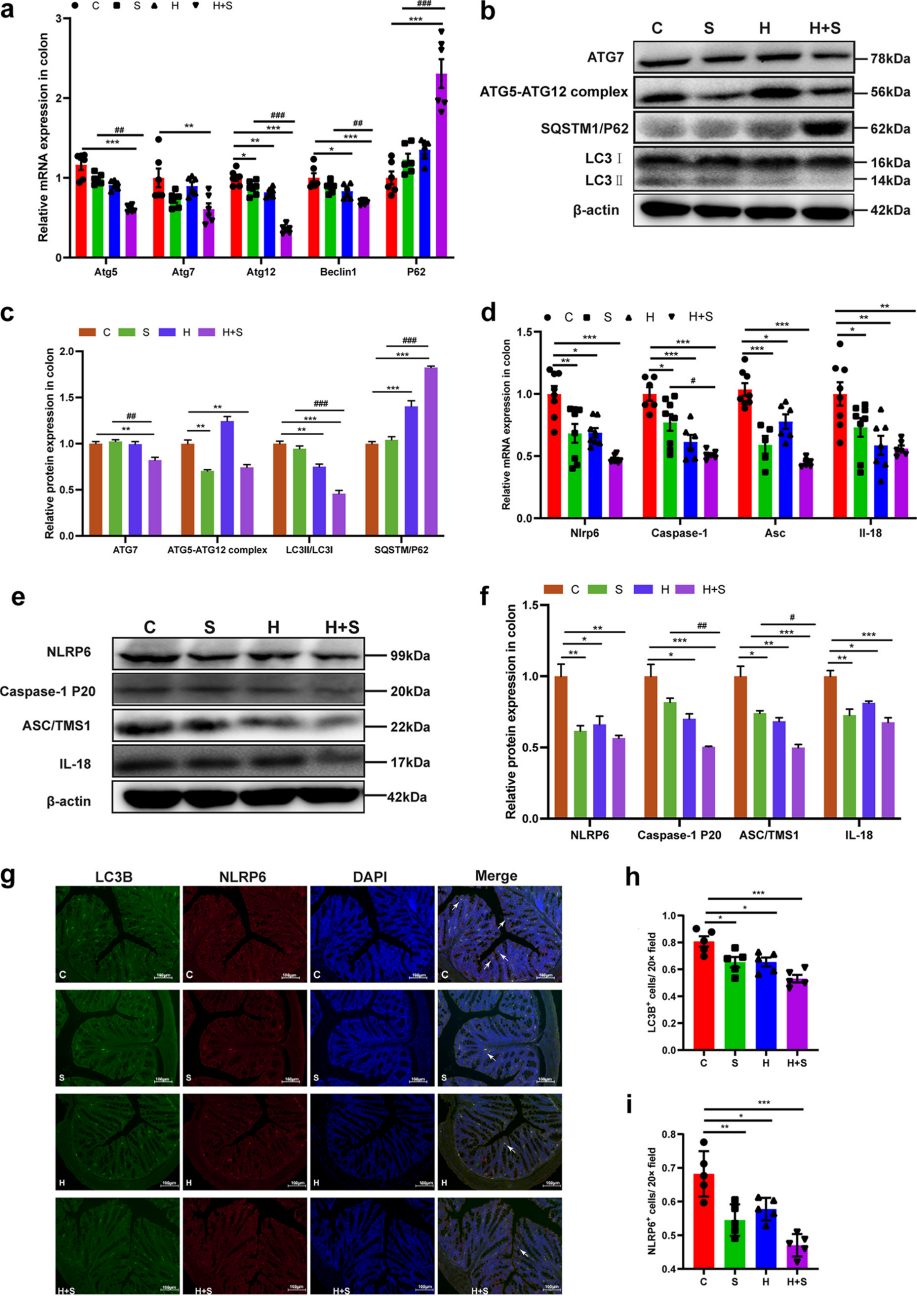
Restraint stress and high-fructose feeding attenuated the occurrence of colonic autophagy and the expression of NLRP6 protein. (a) Changes in mRNA levels of autophagy-related proteins determined by real-time qPCR in mice (*n* = 5 per group). (b and c) The expression of autophagy-related proteins was examined by Western blotting, and relative protein levels were normalized to β-actin (*n* = 5) (c). (d) Changes in mRNA levels of Nlrp6, Asc, caspase-1, and IL-18 by real-time qPCR in mice (*n* = 5 per group). (e and f) The expression of NLRP6-related protein was examined by Western blotting, and relative protein levels were normalized to β-actin (*n* = 5) (f). (g) Representative immunofluorescence image of colonic intestinal epithelium showing a significant reduction of autophagy and NLRP6 levels in the H+S group. Formation of autophagosomes was visualized utilizing the mouse anti-LC3B antibody expression protein (green), and rabbit anti-NLRP6 antibody expression protein (red); epithelial cell nuclei are indicated with DAPI (blue). Bar, 100 μm. (h) Quantitation of autophagosome formation through LC3B-positive cells (green in panel g) in the intestinal epithelium (*n* = 5 per group). (i) Quantification of NLRP6-positive mucin (red in panel g) in intestinal epithelial cells (*n* = 5 per group). Data are means and SEM. *, *P* < 0.05; **, *P* < 0.01; ***, *P* < 0.001 compared with the control group (C). #, *P* < 0.05; ##, *P* < 0.01; ###, *P* < 0.001 compared with the restraint stress group (S). H, high fructose; H+S, high-fructose and restraint stress.

To further verify the influence of these factors on the decrease of goblet cell number and secretion capacity, the proliferation of colon epithelial cells was detected by immunohistochemistry using Ki-67. The IOD of Ki-67-positive cells was significantly lower in the H+S group than in the S group (*P* = 0.000) (Fig. 4a and b). TUNEL (terminal deoxynucleotidyltransferase-mediated dUTP-biotin nick end labeling) staining results showed that the number of apoptotic cells in the H+S group was significantly higher than that in the S group (*P* < 0.001) (Fig. 4a and c). The expression of antiapoptotic protein Bcl-2, apoptotic protein Bax, caspase-3, and poly(ADP-ribose) polymerase (PARP) was detected by Western blotting. The results showed that the expression of Bcl-2 (*P* < 0.001) was significantly inhibited, while the expression of Bax (*P* < 0.001), c-caspase-3 (19 kDa, *P* < 0.001; 17 kDa, *P* < 0.001), and cleaved (c)-PARP (*P* = 0.025) was significantly increased in the H+S group compared with the S group (Fig. 4d and e). The mRNA levels of autophagy-related proteins in H+S group were significantly decreased (Fig. 5a). Western blotting revealed that the expression levels of ATG7 (*P* = 0.003) and LC3II/LC3I (*P* < 0.001) in the H+S group were significantly lower than in the S group, while the expression level of SQSTM1/P62 (*P* < 0.001) in the H+S group was significantly higher than in the S group (Fig. 5b and c). In summary, these results indicated that high fructose intake could aggravate apoptosis and enhance the inhibition of autophagy in colonic tissue under restraint stress.

### High fructose intake could aggravate the inhibition of NLRP6 inflammasome expression under restraint stress conditions.

Next, we investigated the expression level of the NLRP6 inflammasome in the colon. First, RT-PCR results showed that the mRNA levels of Nlrp6 (*P* < 0.001), Asc (*P* < 0.001), caspase-1 (*P* < 0.001), and IL-18 (*P* = 0.005) were significantly inhibited in the H+S group compared with the control group (Fig. 5d). This decrease in the levels of NLRP6-related protein was further confirmed by Western blotting analysis. Likewise, in the H+S group, the protein expression levels of ASC/TMS1 (*P* = 0.016) and caspase-1 (*P* = 0.009) were significantly lower than the S group (Fig. 5e and f). Moreover, we further verified the correlation between the expression of NLRP6 inflammasome and autophagy. We used LC3B and NLRP6 to perform immunofluorescence double-staining experiments. The results showed that decreased levels of LC3B (*P* = 0.000) were localized to colonic NLRP6 (*P* < 0.001) in the H+S group, compared with the control group (Fig. 5g to i). These findings suggested that high fructose intake could disrupt NLRP6 inflammasome signaling under restraint stress conditions, possibly affecting the level of intestinal autophagy.

### High fructose intake promoted gut microbiota disturbances under restraint stress.

**FIG 6:**
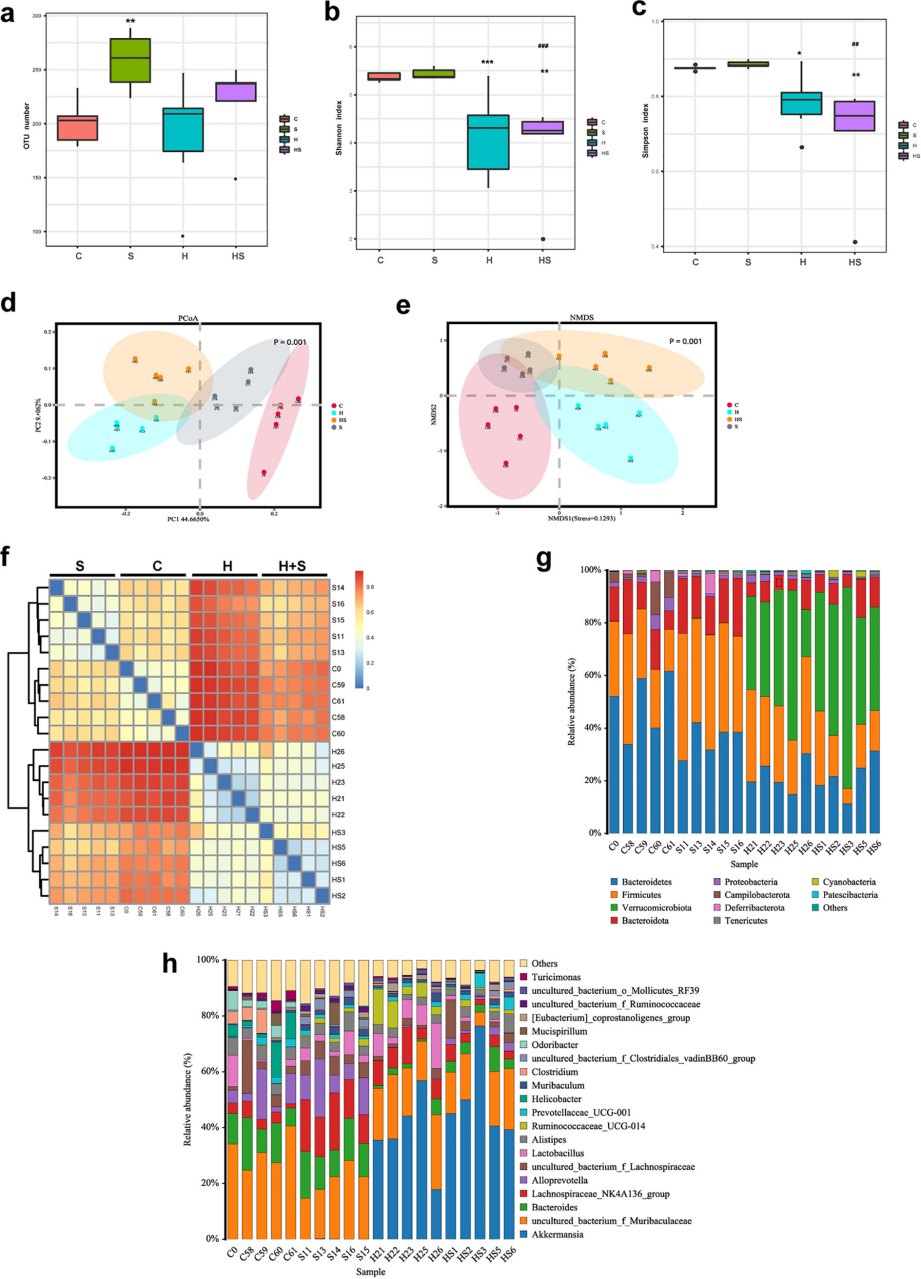
Changes of colonic microbial composition in mice stimulated by restraint stress and high fructose. (a) OTU number in the C, S, H, and H+S groups. The alpha diversity includes diversity and richness. (b and c) Shannon index (b) and Simpson index (c) of the C, S, H, and H+S groups. The beta diversity shows the dispersion of each sample in the four groups. (d and e) PCoA score plot (d) and NMDS (e) score plot based on the Bray-Curtis score plot, which is based on the OTUs in the colon. (f) Species abundance heat map. (g) Species distribution in columnar format, showing the relative contribution of the top 10 phyla in the groups in the colon. (h) Relative abundance of the top 20 genera in the groups in the colon (h). Data are means and SEM. *, *P* < 0.05; **, *P* < 0.01; ***, *P* < 0.001 compared with the control group (C). ##, *P* < 0.01; ###, *P* < 0.001 compared with the restraint stress group (S).

**FIG 7:**
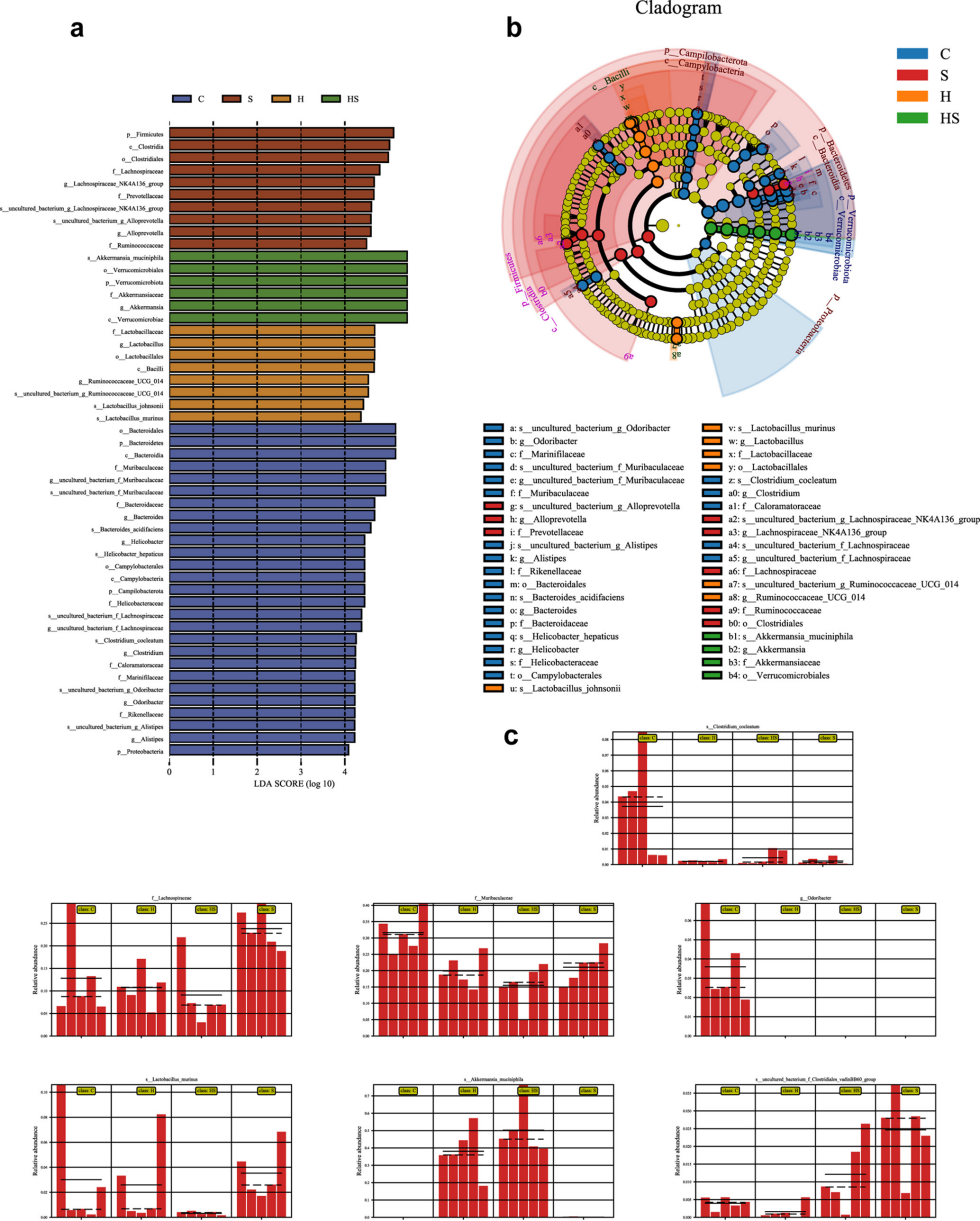
Restraint stress and high-sugar diet led to colonic microbial dysbiosis. (a) The diameter of each circle reflects the abundance of that taxon in the community. Taxa with different abundances in the colons of H+S mice and C, S, and H mice are indicated (*n* = 5). A cutoff value of ≥2.0 was used for LDA. (b) Taxonomic cladogram obtained from LEfSe sequence analysis in the colon. Biomarker taxa are highlighted by colored circles and shaded areas. (c) Relative abundances of Clostridium cocleatum, *Lachnospiraceae*, *Muribaculaceae*, *Odoribacter*, Lactobacillus murinus, Akkermansia muciniphila, and an uncultured bacterium of the *Clostridiales* vadin BB60 group in the colon microbiota based on the LEfSe results. Solid and dashed lines indicate the mean and median, respectively.

The effects of dietary fructose and restraint stress on the colonic microflora of mice were evaluated by 16S rRNA gene amplicon sequencing. The number of operational taxonomic units (OTUs) in the S group was significantly increased (*P* = 0.008), but there was no significant difference among the other groups (Fig. 6a). Analysis of alpha diversity using Shannon and Simpson index, Shannon index (*P* = 0.001 to 0.003) and Simpson index (*P* = 0.014 to 0.003) showed that species richness was significantly reduced in the H and H+S groups (Fig. 6b and c). The Bray-Curtis beta diversity of the four groups of microbial populations and the clustering pattern on principal-coordinate analysis (PCoA) and nonmetric multidimensional scaling (NMDS) plots revealed a distinct clustering of microbiota composition (Fig. 6d and e). The sample heat map analysis by the Bray-Curtis method showed that stress and high-fructose treatment could significantly affect the difference between the microbiotas of the two groups (Fig. 6f). Specifically, we characterized the effect of restraint stress and high fructose on relative abundance of different bacterial taxa at the phylum and genus levels (Fig. 6g and h). We used the linear discriminant analysis (LDA) effect size (LEfSe) algorithm to perform LDA to identify operational microbial taxa that were differentially abundant with regard to restraint stress and fructose intake (Fig. 7a and b). In the H+S group, Clostridium cocleatum, *Lachnospiraceae*, *Muribaculaceae*, *Odoribacter*, and Lactobacillus murinus were among the bacteria that were most extensively reduced, whereas there was increased abundance of *Akkermansiaceae* and an uncultured bacterium of the *Clostridiales* vadin BB60 group in the H+S group (Fig. 7c). Together, these results illustrated that fructose intake could profoundly aggravate the effects of restraint stress on the taxonomic composition of the gut microbiota.

### High fructose intake induced changes in colonic microbiota metabolites stimulated under restraint stress.

**FIG 8:**
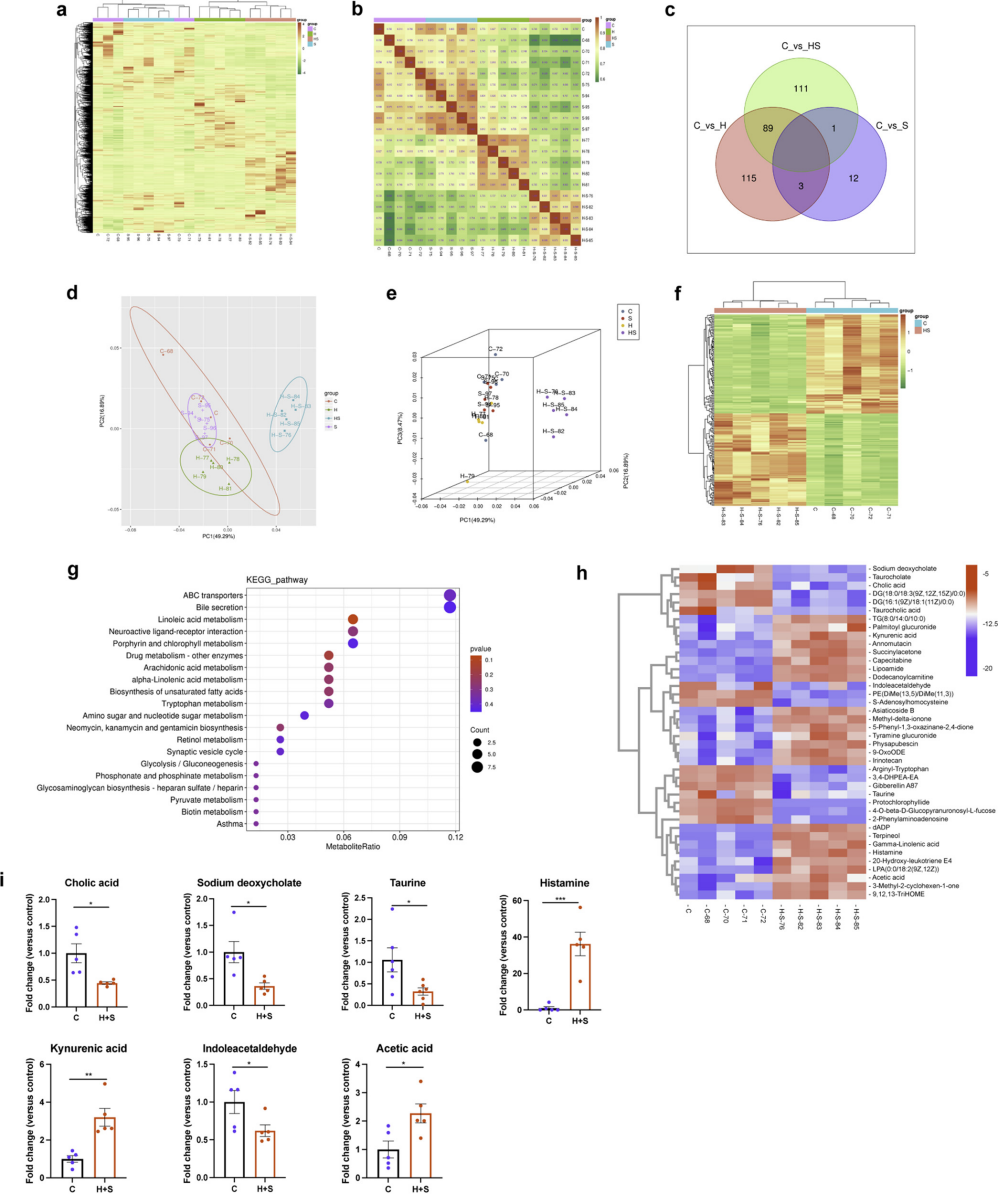
Sequencing analysis of colonic microbiota metabolites. (a) The colonic microbiota metabolites of each group were analyzed by cluster heat map (*n* = 5). (b) Correlation analysis between samples in each group allows assessment of biological duplication between samples within a group (*n* = 5). (c) Venn diagram comparing and analyzing the relationship between different metabolites in each group. (d and e) Principal-component analysis of fecal samples in each group, showing the floor plan and three-dimensional diagram display (*n* = 5). (f) The differential metabolites screened in the C and H+S groups were clustered and analyzed (*n* = 5). (g) KEGG annotation results for different metabolites in the C and H+S groups were analyzed for enrichment. (h) Cluster heat map analysis of the 40 metabolites differentially changed in the C and H+S groups (*n* = 5). (i) Relative abundance of cholic acid, sodium deoxycholate, taurine, histamine, kynurenic acid, indoleacetaldehyde, and acetic acid in the C and H+S groups in the colon microbiota based on the heat map results (*n* = 5). Data are means and SEM.

We further analyzed the changes in microbiota metabolites after stimulation with high fructose and restraint stress. Analysis of the metabolites using an untargeted metabolomics approach showed that there were 2,575 metabolites in the colon. First, cluster heat map analysis of all colonic microbiota metabolites sequenced in the C, S, H, and H+S groups showed obvious differences among the groups, especially after the addition of high fructose (Fig. 8a). Through correlation analysis between samples, it was found that there was a significant correlation between the samples within a group, while the correlation between the samples between groups was not significant, especially when the H+S group was compared with the other groups (Fig. 8b). A Venn diagram showed that different treatments resulted in different metabolite changes, with an additional 115 metabolites increased in the high-fructose group compared with the control group (Fig. 8c). The beta diversity analysis showed an obvious clustering of microbiota metabolite composition in between C and H+S group (Fig. 8d and e). The above results were confirmed again by the cluster heat map analysis of C and H+S groups (Fig. 8f). This suggested that the composition of microbiota metabolites was significantly altered after stimulation with high fructose and restraint stress. To determine how changes in gut microbiota metabolites affect host signaling pathways, we classified the annotated results for differential metabolites in the C versus the H+S group according to pathway type in KEGG. The results showed that bile secretion and tryptophan metabolism pathways were significantly enriched (Fig. 8g). Cluster heat map analysis of the 40 metabolites differentially changed in the C and H+S group (Fig. 8h) revealed that the contents of cholic acid, sodium deoxycholate, taurine, and indoleacetaldehyde (Fig. 8i) were significantly decreased, while the contents of histamine, kynurenic acid, and acetic acid were significantly increased. It could be speculated that high fructose and restraint stress led to changes in metabolites, which in turn affected certain pathways and functions, ultimately leading to intestinal mucosal barrier disruption.

### Taurine promoted expression of MUC2, tight junction, NLRP6, and autophagy proteins in HT-29 cells, while histamine inhibited the expression of NLRP6.

**FIG 9:**
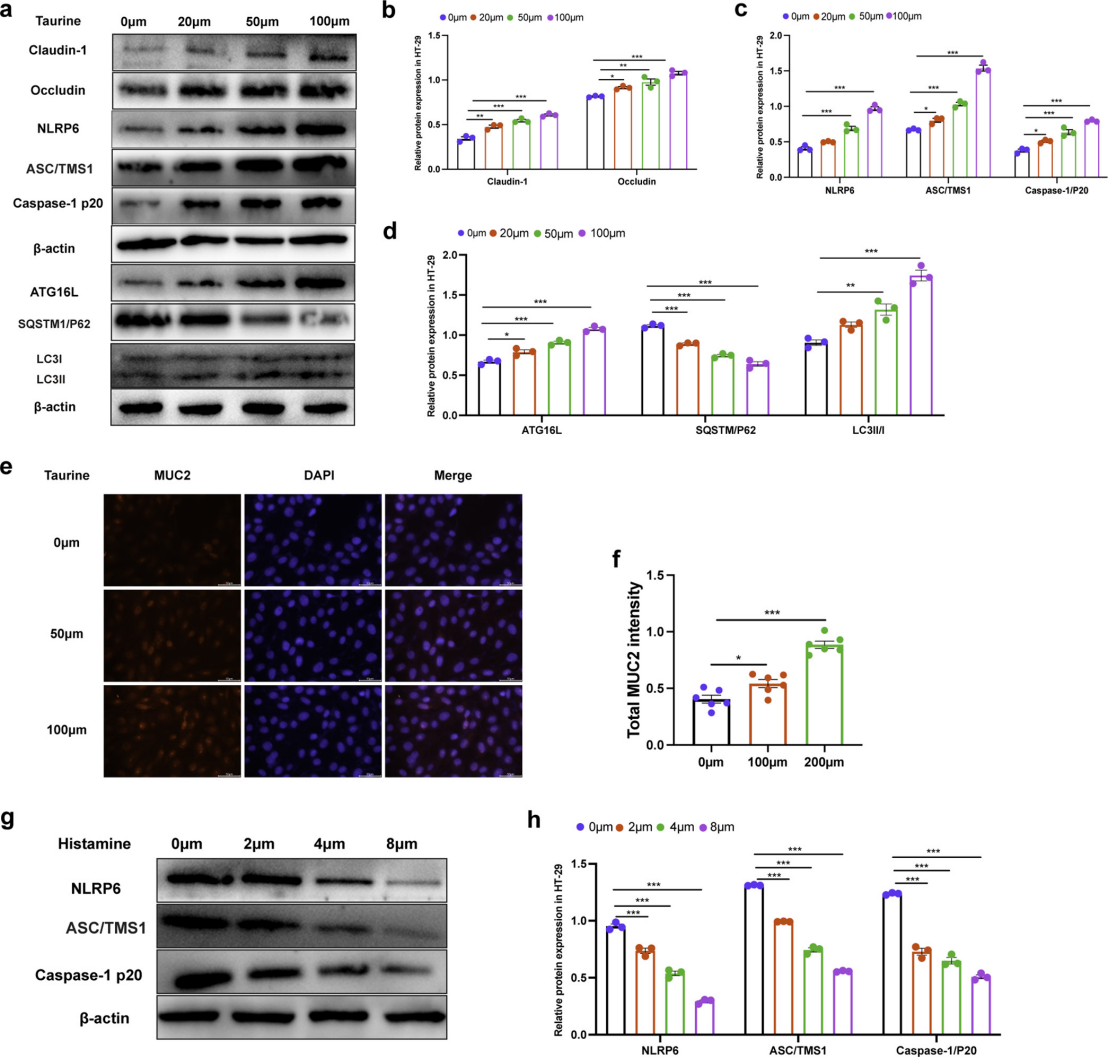
Effects of taurine and histamine on tight junction and MUC2 proteins and on NLRP6 and autophagy proteins, respectively, in HT29 cells. (a) HT-29 cells were treated with different concentrations of taurine. (b to d) The expression of tight junction proteins claudin-1 and occludin, NLRP6, ASC/TMS1, caspase1/p20, ATG16L, SQSTM1/P62, LC3II/I, and β-actin were examined by Western blotting, and relative protein levels were normalized to β-actin (*n* = 3). (e and f) The expression of MUC2 in HT-29 cells treated with different concentrations of taurine was detected by immunofluorescence (e), and MUC2 intensities were analyzed with ImageJ (f) (*n* = 6 hole). (g and h) HT-29 cells were treated with different concentrations of histamine. The expression of NLRP6, ASC/TMS1, caspase 1/p20, and β-actin was examined by Western blotting (g), and relative protein levels were normalized to β-actin (h) (*n* = 3). Data are means and SEM. *, *P* < 0.05; **, *P* < 0.01; ***, *P* < 0.001.

To further understand the effect of microbiota-modulated metabolites on the intestinal mucosal barrier, we hypothesized that the changes in the abundance of histamine and taurine affect regulation of MUC2, tight junction protein, NLRP6, and autophagy protein expression. Different concentrations of taurine were added to the medium and incubated with HT-29 cell monolayers. Interestingly, taurine supplementation significantly increased the expression of the tight junction protein claudin-1 (*P* < 0.001) and occludin (*P* < 0.001) (Fig. 9a and b) and promoted the expression of NLRP6 (*P* < 0.001), caspase-1 p20 (*P* < 0.001), and ASC/TMS1 (*P* < 0.001) (Fig. 9a and c) and autophagy proteins ATG16L (*P* < 0.001), SQSTM1/P62 (*P* < 0.001), and LC3II/I (*P* < 0.001) (Fig. 9a and d) in a dose-dependent manner, compared with the control group. The results of MUC2 immunofluorescence showed that taurine (50 μM and 100 μM) supplementation could promote the expression of MUC2 protein in TH-29 cells (Fig. 9e and f). The above results indicated that taurine enhanced the integrity of the intestinal mucosal barrier by increasing the expression of tight junction and MUC2 proteins and also significantly increased the expression of NLRP6 and autophagy protein. However, histamine supplementation in HT-29 cells inhibited NLRP6 (*P* < 0.001), caspase-1 p20 (*P* < 0.001), and ASC/TMS1 (*P* < 0.001) expression in a dose-dependent manner, compared with the control group (Fig. 9g and h). This suggested that high fructose- and restraint stress-induced intestinal mucosal barrier damage may be affected by changes in taurine and histamine levels.

### Intestinal colonization with *A. muciniphila* attenuated colonic barrier damage from high-fructose and restraint stress stimuli.

**FIG 10:**
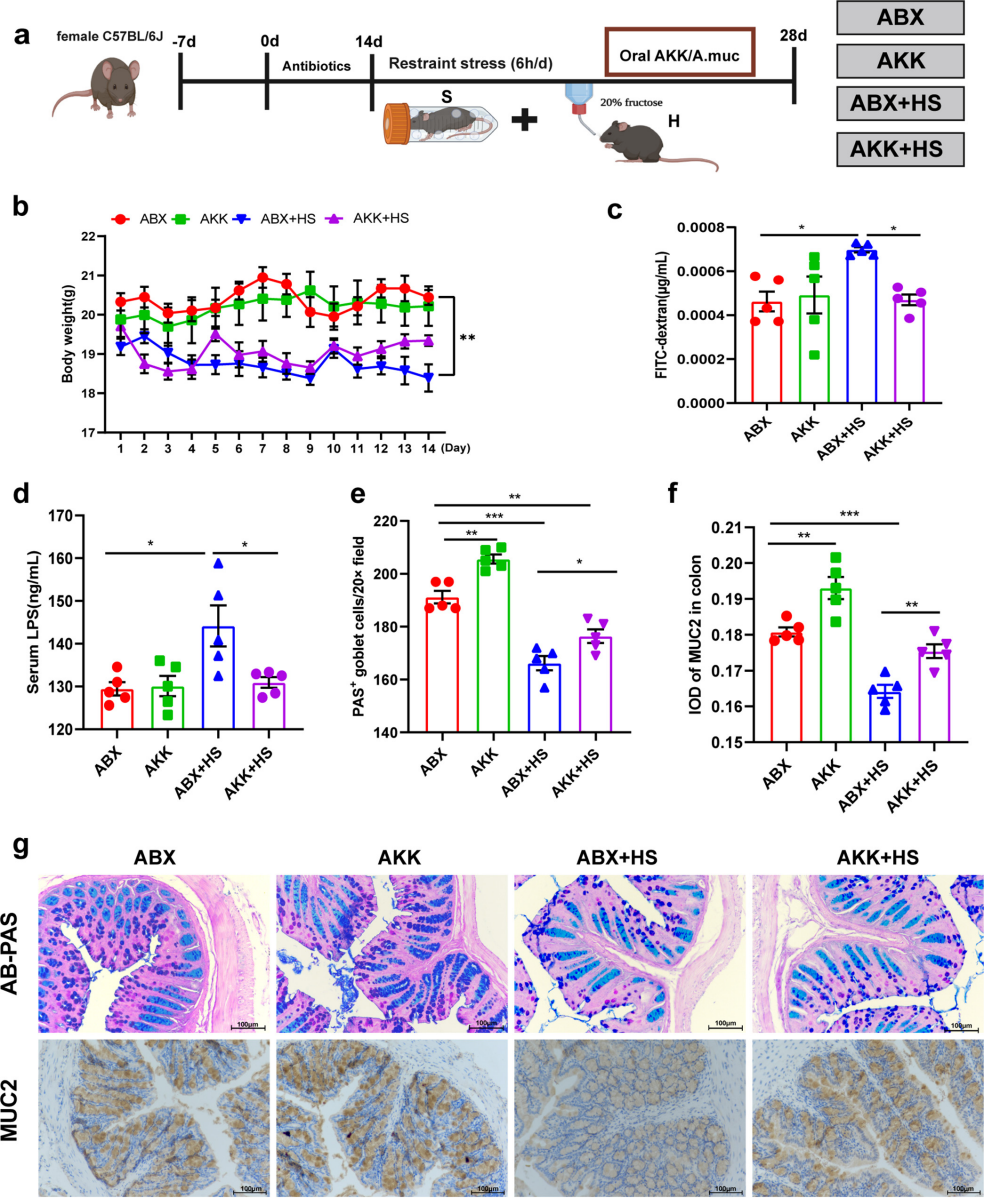
After colonization of *A. muciniphila* in mice, the colonic mucosal barrier function was enhanced. (a) Study design of experiment II. (b) Daily body weight. (c) Analysis of intestinal permeability using FITC-dextran in serum. Mice received FITC-dextran by gavage. Serum samples were collected 4 h later (*n* = 5). (d) Serum endotoxin levels were determined by ELISA. (e) Enumeration of goblet cells. (f) IOD of MUC2 in the intestinal tissue from each treatment group (*n* = 5; 20 microscopic images were obtained from each treatment group). (g) AB-PAS-stained mouse colon sections showing the goblet cells and immunohistochemistry staining of MUC2 in intestinal tissue sections. Bars, 100 μm. Data are means and SEM. *, *P* < 0.05; **, *P* < 0.01; ***, *P* < 0.001 compared with the control group (C).

Next, we further explored the effect of *A. muciniphila* colonization on intestinal mucosal immunity (Fig. 10a). First, we determined changes in fecal flora abundance; the results showed that the abundance of *Bacteroides*, *Firmicutes*, *Lactobacillus* and *A. muciniphila* decreased significantly in the antibiotic treatment (ABX) group (*P* < 0.001) and the H+S group receiving antibiotic treatment (ABX+HS group) (*P* < 0.001) (Fig. S3A to D). In the *A. muciniphila* colonization (AKK) group (*P* < 0.001) and AKK+HS group (*P* < 0.001), the abundance of *Bacteroides*, *Firmicutes*, and *Lactobacillus* decreased significantly, while the abundance of *A. muciniphila* increased significantly (*P* < 0.001) (Fig. S3E to H), indicating that the antibiotic treatment and *A. muciniphila* colonization were successful.

**FIG 11:**
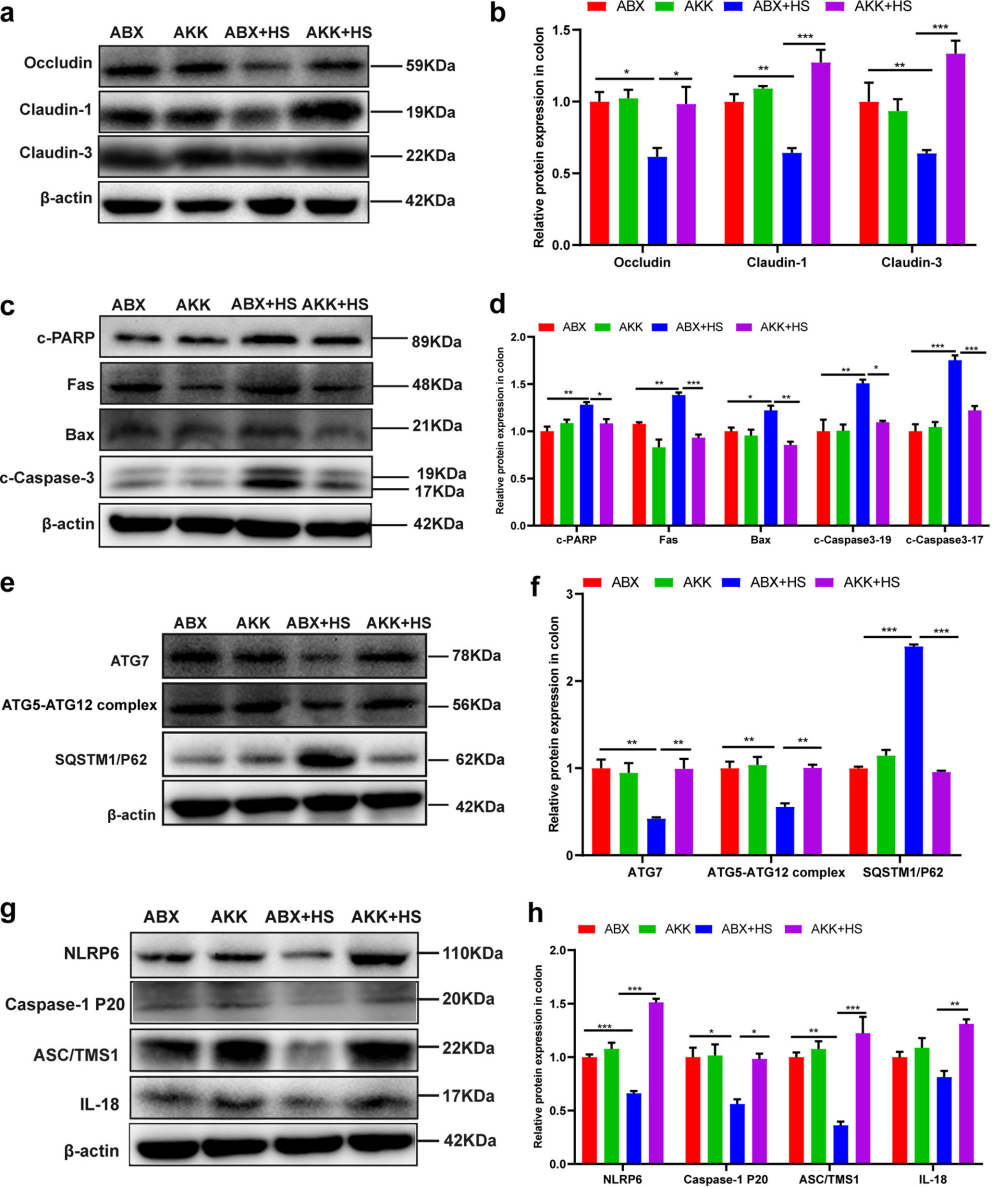
Colonization of *A. muciniphila* in mice reduced colonic apoptosis, increased autophagy, and promoted the expression of NLRP6 inflammasome. (a and b) The expression of colon tight junction proteins occludin, claudin-1, and claudin-3 and β-actin were examined by Western blotting (a), and relative protein levels were normalized to β-actin (*n* = 5) (b). (c and d) Production of apoptotic protein Fas, Bax, caspase-3, and PARP was examined by Western blotting (c), and relative protein levels were normalized to β-actin (*n* = 5) (d). (e) The expression of autophagy related proteins were examined by Western blotting, and relative protein levels were normalized to β-actin (*n* = 5) (f). (g and h) The expression of NLRP6-related protein was examined by Western blotting, and relative protein levels were normalized to β-actin (*n* = 5) (h). Data are means and SEM. *, *P* < 0.05; **, *P* < 0.01; ***, *P* < 0.001.

Animals exposed to high fructose and restraint stress did not experience significant body weight (*P* = 0.122) changes after *A. muciniphila* colonization. In contrast, the antibiotic-treated mice significantly lost weight (*P* = 0.002) after being stimulated by high fructose and restraint stress (Fig. 10b). Interestingly, there were no changes in the livers and spleens of the antibiotic-treated and *Akkermansia*-colonized mice (Fig. S1C and D). Intestinal permeability was significantly lower in the AKK+HS group (*P* = 0.023) than the ABX+HS group (Fig. 10c). Serum endotoxin levels were consistent with gut permeability results showing that the endotoxin level in the AKK+HS group (*P* = 0.022) were significantly lower than in the ABX+HS group (Fig. 10d). AB-PAS staining showed that the number of colonic goblet cells was significantly increased after *A. muciniphila* colonization, and the staining of mucin in crypts was significantly deepened (Fig. 10e and g). Similarly, immunohistochemical results of MUC2 showed that mucin secretion capacity in the AKK+HS group was significantly higher than in the ABX+HS group (*P* = 0.009) (Fig. 10f and g). The expression of colonic tight junction protein was detected by Western blotting. The results showed that the expression of occludin (*P* = 0.047), claudin-1 (*P* = 0.000), and claudin-3 (*P* = 0.001) in the AKK+HS group was significantly higher than in the ABX+HS group (Fig. 11a and b). These data showed that high fructose combined with restraint stress stimulation after colonization with *A. muciniphila* reduced the damage to the intestinal barrier.

### Intestinal colonization with *A. muciniphila* reduced the level of apoptosis and restored the level of autophagy and the expression of NLRP6 induced by high fructose and restraint stress.

The expression of apoptosis, autophagy, and NLRP6-related proteins in the colon was detected by Western blotting. We found that levels of the apoptosis protein c-PARP (*P* = 0.034), Fas (*P* = 0.001), Bax (*P* = 0.003), and c-caspase-3 (19 kDa, *P* = 0.016; 17 kDa, *P* = 0.001) in the AKK+HS group were significantly lower than in the ABX+HS group (Fig. 11c and d). The expression of autophagy protein ATG7 (*P* = 0.007) and the ATG5-ATG12 complex (*P* = 0.005) increased significantly while the expression of SQSTM1/P62 (*P* < 0.001) decreased in the AKK+HS group compared with the ABX+HS group (Fig. 11e and f). The expression of the inflammasome 6-related protein NLRP6 (*P* < 0.001), caspase-1 (*P* = 0.017), ASC/TMS1 (*P* = 0.001), and IL-18 (*P* = 0.002) in the AKK+HS group was significantly higher than in the ABX+HS group (Fig. 11g and h). These data demonstrated that *A. muciniphila* colonization reversed the abnormal levels of colonic apoptosis, autophagy, and NLRP6 induced by high fructose and restraint stress.

## DISCUSSION

A number of epidemiological, observational, prospective, and retrospective case-control studies have examined the impact of diet as a risk factor for the development of IBD and IBS (25, 26). Chronic intake of fructose has been shown to be associated with a loss of tight junction protein in the intestine as well as an increased translocation of bacterial endotoxins from the intestine to the liver (27). It has also been found that intake of high fructose promotes colitis pathogenesis (28). Although it has been predicted that a high-fructose diet is a key contributor to the development of IBD, the exact mechanism remains unclear. This study using an animal model provides compelling evidence that a fructose-rich diet predisposes individuals to the pathogenesis of inflammatory bowel diseases.

In the present study, using a murine restraint model to simulate the occurrence of intestinal inflammation, we showed that stress could alter intestinal host-commensal homeostasis and induce mucosal damage and microbial dysbiosis in the gut. These data are consistent with prior publications on stress-induced disorders of the intestinal microbiota and immune disorders (3, 29, 30). However, the intake of fructose could aggravate the occurrence and development of these symptoms. Moreover, the present findings showed that high-fructose intake combined with restraint stress could enhance oxidative stress and proinflammatory reactions. Substantial evidence supports the coupling of increased oxidative stress with chronic intestinal inflammation (31).

The colonic mucus layer is an early barrier that pathogenic microorganisms must transit to enter the intestinal lumen (32). When this barrier is breached, it increases susceptibility to pathogens and the risk of inflammatory bowel disease (12, 22). We observed that the normal histological composition of colon tissues was destroyed and goblet cell number and mucin secretion capacity were significantly reduced in the H+S group. The mechanism by which host immunity is involved in the microbial interface encompasses IgA and mucous secretion as well as antimicrobial-peptide (AMP) production (33). We found that the expression of antimicrobial peptides and SIgA was significantly reduced in the H+S group. Tight junction protein expression affects the integrity and permeability of intestinal mucosa (34). Therefore, in line with our expectations, excessive fructose ingestion caused weakening of tight junction protein expression, which worsened the stress-induced barrier deterioration. As demonstrated by Todoric et al. (27) and Do et al. (35), excessive fructose intake leads to downregulation of tight-junction proteins, intestinal barrier deterioration, and endotoxemia.

In this study, we further analyzed the mechanism of feeding high levels of fructose to mice stimulated by restraint stress affecting intestinal mucosal injury. Previous studies have found that intestinal goblet cells secrete mucus that requires autophagy (36). Several studies have reported changes in granular structure in secretory IEC with autophagy defects, such as goblet cells (37, 38). This indicates that autophagy is necessary for goblet cells to maintain normal secretory function. NLRP6 is a key regulator of colon homeostasis (16, 39). NLRP6 is expressed primarily in intestinal epithelial cells, including goblet cells, where it has been found to be essential for mucosal self-renewal, proliferation, and mucus secretion (16, 39). Moreover, studies have found that NLRP6-deficient mice are susceptible to *Citrobacter* infection as a result of altered secretion of goblet cell mucus particles due to impaired autophagy (39). A study by Xiao et al. found that NLRP6 is involved in inflammation and brain damage after cerebral hemorrhage by activating autophagy (40). These results suggested that the NLRP6 inflammasome plays an important role in the regulation of autophagy activation. In our study, colon NLRP6 and autophagy-related proteins were significantly downregulated, and the number of goblet cells and the ability to secrete MUC2 were significantly reduced, which damaged the intestinal mucosal barrier, in the H+S group. These results suggested that NLRP6 expression deficiency may affect the normal autophagy function of goblet cells, leading to the weakened mucin secretion ability. However, the exact mechanisms by which NLRP6 expression regulates autophagy remain an enigma.

Intriguingly, another study reported that normal expression of NLRP6 could maintain intestinal microenvironment homeostasis (15). Therefore, microbial composition was analyzed further in this study. The proportion of pathogenic bacteria of the *Clostridiales* vadin BB60 group, which is a Gram-positive intestinal pathogen that can cause colitis and diarrhea upon treatment with antibiotics (41), was increased in the H+S group. The abundance of Clostridium cocleatum decreased significantly in our study; according to reports of patients with IBS, the level of Clostridium cocleatum decreases (42). The abundances of *Muribaculaceae* and *Odoribacter*, which are butyrate-producing bacteria (43, 44), were significantly reduced in the H+S groups; butyric acid can accelerate the repair of intestinal epithelial cell injury and maintain intestinal homeostasis (45). The relative abundances of *Lachnospiraceae* and *Lactobacillus* were reduced in the H+S group. *Lachnospiraceae* has an anti-inflammatory effect, while its reduced content suggests that it is associated with susceptibility to colitis (46). *Lactobacillus* could induce the expression of anti-inflammatory cytokines and prevent the colonization of pathogens into the intestinal epithelium, thus maintaining intestinal homeostasis (47).

The abundance of *A. muciniphila*, a member of the *Verrucomicrobia*, increased significantly through high-fructose feeding. Similar results were observed with dietary simple sugars, which increased *A. muciniphila* and promoted colitis in mice (28). In addition, the diversity and richness of intestinal flora in NLRP6-deficient C57BL/6 mice were significantly reduced, but the abundance of *A. muciniphila* was significantly increased (48). Unexpectedly, by colonizing mice with *A. muciniphila*, we found that stimulation with high fructose and restraint stress did not damage the intestinal barrier, and changes in intestinal permeability and serum endotoxin levels were mainly absent. An explanation for the counterintuitive lack of a direct relationship between serum endotoxin levels and the abundance of Gram-negative bacteria (Akkermansia muciniphila), suggested by Everard et al., is that Akkermansia muciniphila regulates barrier function at different levels of the gut (21). The colonization by *A. muciniphila* increased the number of mucus-producing goblet cells in the colon, consistent with previous reports (49). At the same time, the expression of tight junction protein was significantly increased. These data were consistent with *A. muciniphila* colonization increasing goblet cell numbers and upregulating tight junction protein expression in obese mice and mice with alcoholic fatty liver (21, 50). Recent studies have shown that extracellular vesicles from *A. muciniphila* MucT (AmEVs) reduce intestinal permeability by regulating tight junctions in mice (51). Notably, *Akkermansia*-colonized mice were able to restore intestinal autophagy levels and NLRP6 expression and reduce the occurrence of intestinal apoptosis after stimulation with high fructose and restraint stress. However, whether this was directly regulated by *A. muciniphila* or through its specific microbiota metabolites remains unclear.

Metabolites are thought to be key mediators of host-microbiome communication (52). Here, we observed a significant decrease in cholic acid, indoleacetaldehyde, and taurine in the colons of the H+S group. Studies have found that cholic acid can be produced to indirectly stimulate host production of antimicrobial peptides, such as cathelicidin and angiogenin I (53). Indoleacetaldehyde is a tryptophan metabolite that play a critical role in maintaining the homeostasis of the gut and systematic immunity (54). Recent studies have shown that upregulation of taurine in the gut increases intestinal epithelial integrity by strengthening tight junctions to reduce intestinal leakage and inflammation (55). Other independent studies have also shown that the addition of taurine to drinking water reduces dextran sulfate sodium-induced colitis (56). We found that *in vitro*, taurine could promote the expression of tight junction protein and the mucin MUC2, confirming that taurine can enhance the integrity of the intestinal mucosal barrier. In addition, taurine was found to be a microbe-dependent positive inflammasome modulator that enhanced NLRP6 inflammasome function (15). Recently, taurine was found to alleviate inflammation caused by Streptococcus uberis by activating autophagy in mammary epithelial cells (57). Interestingly, these observations are consistent with our *in vitro* results that taurine treatment of HT-29 cells promoted NLRP6 and autophagy protein expression. The abundance of kynurenic acid increased in the H+S group, and it has been found to bind to the aryl hydrocarbon receptor (AhR) to produce the proinflammatory cytokine IL-6 (58). Moreover, the highest increase in histamine levels was in the colons of the H+S group. The microbial metabolite histamine has been found to inhibit NLRP6 expression (15, 59), which is consistent with our *in vitro* results. Therefore, these results suggested that microbe-host interactions may directly affect intestinal mucosal barrier development through microbiota metabolites. However, the exact mechanism by which taurine depletion and histamine increase lead to these dysfunctions and the related signaling pathways remain largely unknown. In conclusion, the intestinal microbiota and microbiota metabolites play an important role in maintaining homeostasis of the intestinal mucosal barrier microenvironment. At the same time, this study provides novel avenues to explore and develop therapeutic options for intestinal diseases in the future.

### Conclusion.

**FIG 12:**
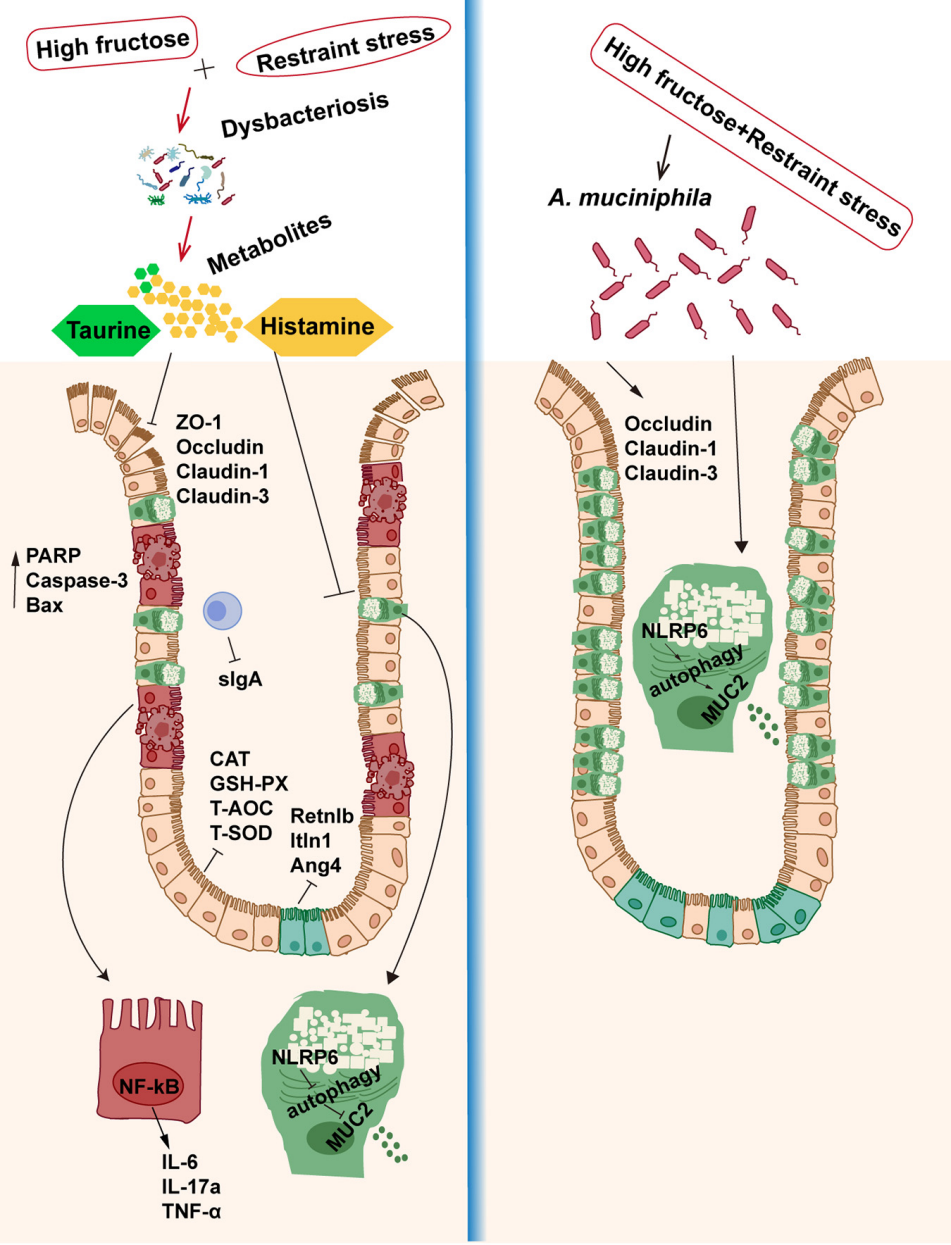
The disruption of the intestinal mucosal barrier induced by high fructose and restraint stress was regulated by intestinal microbiota and microbiota metabolites. Dietary fructose exacerbated the development of restraint stress, a strong change in the composition of the gut microbiota and microbial metabolites, decreasing taurine abundance, increasing histamine abundance, increasing intestinal permeability, decreasing expression levels of tight junction proteins, and limitation of the expression of NLRP6 inflammasomes, thereby affecting the normal autophagy level and disrupting the function of colonic goblet cells to secrete mucus, leading to the destruction of the intestinal mucosal immune barrier, activating the NF-κB signaling pathway and inducing an intestinal inflammatory response. However, *A. muciniphila* supplementation counteracted damage to the intestinal mucosal barrier caused by high fructose and restraint stress. The colonization of *A. muciniphila* could increase the expression of colonic tight junction protein, restore the function of NLRP6 inflammasomes, activate autophagy, and promote the ability of goblet cells to secrete mucin, thereby protecting the intestinal mucosal barrier and reducing the occurrence of intestinal inflammation.

High-fructose consumption exacerbated alterations in the composition of the intestinal microbiome and microflora metabolites caused by restraint stress, leading to an increase in intestinal permeability and a decrease in NLRP6 and autophagy protein expression, destroying the function of colonic goblet cells, increasing intestinal inflammation, and leading to intestinal mucosal barrier defects. However, these phenomena were reversed when mice were colonized with *A. muciniphila*, and a possible mechanism is shown Fig. 12. These results show that the intestinal microbiome and microbiota metabolites play an important role in maintaining intestinal mucosal barrier microenvironment homeostasis and that *A. muciniphila* may be able to be used in the treatment of intestinal diseases.

## MATERIALS AND METHODS

### Animal treatments.

A total of 80 female C57BL/6J mice (5 to 6 weeks old) were purchased from Vital River Laboratory Animal Technology Co., Ltd. (Beijing, China), and housed under specific-pathogen-free (SPF) conventional conditions (14 h daylight per cycle). After 1 week of adaptation, the animals were divided for two types of animal experiments. In experiment I (Fig. 1a), 40 mice were randomly assigned to four groups: the control group (C; *n* = 10), the restraint stress group (S; *n* = 10), the high-fructose group (H; *n* = 10), and the group receiving high fructose and undergoing restraint stress (H+S; *n* = 10). Mice were individually placed into ventilated transparent 50-mL plastic centrifuge tubes to limit their movements for 6 h (from 10:00 to 16:00) for 14 consecutive days in the S and H+S groups. Then, 20% fructose was added to the drinking water of the mice in the H and H+S groups. During the restraint stress period, the mice in the control group and the high-fructose group were not given food and water.

In experiment II (Fig. 8a), 40 mice were randomly assigned to four groups: the antibiotic treatment group (ABX; *n* = 10), the *A. muciniphila* colonization group (AKK; *n* = 10), the group receiving antibiotic treatment and high fructose and undergoing restraint stress (ABX+HS; *n* = 10), and the group receiving *A. muciniphila* colonization and high fructose and undergoing restraint stress (AKK+HS; *n* = 10). Antibiotic treatment was given in the drinking water. The antibiotics were formulated as 1 g/L ampicillin, 100 mg/L gentamicin, 0.5 g/L neomycin, 0.5 g/L vancomycin, and 10 mg/L erythromycin, given continuously in drinking water for 14 days. All antibiotics were obtained from Solarbio Science & Technology Co., Ltd. (Beijing, China). After antibiotic treatment ended, in the AKK and AKK+HS groups, mice were treated with *A. muciniphila* by oral gavage at a dose of 1 × 10^8^ CFU/0.2 mL, suspended in sterile anaerobic phosphate-buffered saline (PBS). *A. muciniphila* was given via intragastric administration before constraint stress. High-fructose and restraint stress treatments were the same as in experiment 1. The control group was orally administered an equal volume of sterile anaerobic PBS containing similar final concentrations of glycerol.

At the end of the treatment, the mice were euthanized for cervical dislocation, followed by decapitation to immediately collect trunk blood, intestinal tissues, and colonic content. Intestinal segments were fixed in 4% paraformaldehyde. Tissue samples were rapidly frozen and stored in liquid nitrogen for molecular analyses.

### Ethics approval and consent to participate.

All animal experiments were approved by the Institutional Animal Care and Use Committee of the China Agricultural University, Beijing, China, under permit no. AW11011202-2-1 (Beijing, China). In this study, all experimental methods were performed following the China Agricultural University of Health Guide for the Care and Use of Laboratory Animals.

### Endotoxin levels.

Endotoxin levels in mouse blood were determined using an ELISA-based method (CK-E20316; Laibo Terui Technology Development Co., Ltd., Beijing, China) per the manufacturer’s instructions. The concentrations of endotoxin were expressed in nanograms per milliliter of plasma.

### Blood glucose.

Blood glucose levels in the tail blood were measured by a Roche blood glucose meter (GC14906201; Accu-Chek Active; Roche, Germany) before sacrifice.

### Measurement of serum CORT and NE.

The total CORT and NE concentrations were detected by using a mouse CORT and NE radioimmunoassay kit (HY-114; HY-169; Laibo Terui Technology Development Co., Ltd., Beijing, China). All tests were performed according to the kit’s instructions.

### Intestinal permeability.

An *in vivo* permeability test was performed using the fluorescein isothiocyanate (FITC)-labeled dextran method to evaluate the barrier function. Food and water were withdrawn overnight, and mice were subjected to gavage with 60 mg FITC-labeled dextran per 100 g body weight (46944; Sigma-Aldrich Co., Ltd., Shanghai, China). Serum was collected 5 h after FITC–dextran-4 (FD-4) gavage, and the fluorescence intensity of each sample was measured (excitation, 492 nm; emission, 525 nm). FITC-dextran was diluted in phosphate-buffered saline (PBS) to form a standard curve. FITC-dextran concentration in serum was calculated using a standard curve.

### SIgA measurement.

Intestinal tissue and normal saline were ground in a ratio of 1:9, and then the supernatant was taken by centrifugation. SIgA concentrations were measured using competitive ELISA kits (CK-E20416; Laibo Terui Technology Development Co., Ltd., Beijing, China). The concentrations of SIgA were expressed in nanograms per milliliter. All assays were performed according to the kit manufacturer’s instructions.

### Quantitative real-time PCR.

Total RNA was extracted with TRIzol reagent (CW0580; CoWin Biotech Co., Inc., Beijing, China). The tissue samples were ground to powder form with liquid nitrogen, transferred to an RNase-free centrifuge tube containing TRIzol, and mixed by shock. Samples were let stand at room temperature for 5 min to allow the RNA to be fully released. Chloroform was added, and mixtures were shaken vigorously and left at room temperature. Samples were then centrifuged, and the supernatant was removed. The same volume of isopropyl alcohol was added, and mixtures were left at room temperature. Mixtures were centrifuged; the supernatant was discarded, and the precipitate was kept. Ethanol (75%) was added and mixed by rotation or reverse mixing; mixtures were then centrifuged, and the supernatant was discarded. The precipitate was left at room temperature and dried with ethanol, and an appropriate amount of RNase-free water was added to dissolve RNA. Mixtures were again let sit at room temperature until the RNA was completely dissolved. The concentration and purity of RNA were measured with a NanoPhotometer (P330; Implen, Germany). Then, cDNA was synthesized with HiScript QRTsupermix for quantitative qPCR (+gDNA wiper) (R312-02; Vazyme Biotech Co., Ltd., Naijing China). Real-time qPCR was performed with SYBR green master mix (Q141-02; Vazyme Biotech Co., Ltd., Naijing China). Changes in fluorescence were monitored on a OneStep Plus instrument (Applied Biosystems, USA). The primers used are shown in Table 1.

**TABLE 1:** Primers for real-time PCR

Gene product	Primer sequence (5′–3′)		Source
	Forward	Reverse	
Muc2	AGGGCTCGGAACTCCAGAAA	CCAGGGAATCGGTAGACATCG	NM_023566.4
Tff3	TTGCTGGGTCCTCTGGGATAG	TACACTGCTCCGATGTGACAG	NM_011575.2
Klf3	GAAGCCCAACAAATATGGGGT	GGACGGGAACTTCAGAGAGG	XM_006503751.5
Lgr5	CAGCCTCAAAGTGCTTATGCT	GTGGCACGTAACTGATGTGG	XM_021173502.2
Olfm4	CAGCCACTTTCCAATTTCACTG	GCTGGACATACTCCTTCACCTTA	XM_021204082.2
Itln1	TGACAATGGTCCAGCATTACC	ACGGGGTTACCTTCTGGGA	XM_029475723.1
Retnlb	AAGCCTACACTGTGTTTCCTTTT	GCTTCCTTGATCCTTTGATCCAC	XM_021185515.1
Ang4	GGTTGTGATTCCTCCAACTCTG	CTGAAGTTTTCTCCATAAGGGCT	XM_021154346.1
Atg5	TGTGCTTCGAGATGTGTGGTT	ACCAACGTCAAATAGCTGACTC	NM_053069.6
Atg7	TGACCTTCGCGGACCTAAAGA	CCCGGATTAGAGGGATGCTC	NM_028835.5
Atg12	GAAGGCTGTAGGAGACACTCCT	GGAAGGGGCAAAGGACTGATTC	NM_026217.3
Beclin1	ATGGAGGGGTCTAAGGCGTC	TCCTCTCCTGAGTTAGCCTCT	NM_019584.4
P62	GAACTCGCTATAAGTGCAGTGT	AGAGAAGCTATCAGAGAGGTGG	NM_011018.3
Nlrp6	TGACCAGAGCTTCCAGGAGT	TTTAGCAGGCCAAAGAGGAA	XM_006536149.4
ASC	ACAGAAGTGGACGGAGTGCT	CTCCAGGTCCATCACCAAGT	NM_023258.4
Caspase-1	CACAGCTCTGGAGATGGTGA	CTTTCAAGCTTGGGCACTTC	XM_021172833.1
IL-18	GACAGCCTGTGTTCGAGGAT	TGGATCCATTTCCTCAAAGG	XM_036154619.1

### Measurements of antioxidant activity and lipid peroxidation.

The intestinal segments were weighed and placed in a 0.9% saline solution (1:9) to prepare a homogenate. The supernatant was extracted by centrifugation (2,000 × *g*, 20 min). The total protein concentration was measured using a bicinchoninic acid (BCA) protein assay kit (CW0014S; CoWin Biotech Co., Inc., Beijing, China). To assess antioxidant capacity, the activities of superoxide dismutase (Total-SOD) (A001-1), catalase (CAT) (A007-1), and glutathione peroxidase (GSH-Px) (A005-1), as well as total antioxidant capability (T-AOC) (A015-2) and malondialdehyde (MDA) (A003-1) levels, were quantified using colorimetric methods from the Nanjing Jiancheng Bioengineering Institute (Nanjing, China). Except for MDA, which is expressed in micromoles per milligram of protein, the values are expressed in units per milligram of protein. These results were detected at specific wavelengths (SOD, 550 nm; CAT, 405 nm; GSH-Px, 412 nm; T-AOC, 520 nm; and MDA, 532 nm).

### Microbial DNA extraction and full-length 16S rRNA gene sequencing.

The bacterial genomic DNA was isolated from frozen colon contents according to the manufacturer’s instructions by using a PowerSoil DNA isolation kit (MoBio, Shanghai, China). The purity and concentration of the obtained DNA were determined by means of a Nanodrop 1000 instrument (Thermo Fisher Scientific, Wilmington, DE, USA). The preparation of the amplified library uses PCR to amplify the full length of the 16S rRNA. All amplicon libraries were sequenced using a PacBio SMRT platform (Pacific Biosciences, Menlo Park, CA, USA). The bioinformatics analysis of this study was performed with the aid of the BMK Cloud platform (Biomarker Technologies Co., Ltd., Beijing, China).

The UCHIME algorithm (28) (V4.2) was used to detect and remove the chimeric sequence to obtain clean reads. USEARCH (V10.0) was used to cluster sequences with a similarity of 97% into the same operational taxonomic unit (OTU), and the OTUs with abundances of <0.005% were filtered (24). The abundances of OTUs were normalized with the serial number standard corresponding to the sample with the least sequence, and alpha diversity and beta diversity were further analyzed based on the normalized output data. QIIME (V1.8.0), mothur (V1.3.0), and R software (V3.1) were used for alpha diversity analysis, including sparseness, Shannon curve, and Shannon and Simpson calculations. Beta diversity was calculated by QIIME (V1.8.0) using weighted and unweighted UniFrac distance matrices, including PCA and NMDS heat maps (25). LEfSe analysis was performed to find biomarkers with statistical differences between groups (26). Simply put, LEfSe analysis was done with an LDA threshold of >4, using nonparametric factor Kruskal-Wallis (KW) and rank tests and then using the (unpaired) Wilcoxon rank sum test to identify the most diverse taxa.

### Metabolomic analysis.

Fecal samples were added to the extract (volume ratio of methanolic acetonitrile to water, 2:2:1; internal standard concentration, 2 mg/L), vortexed, and mixed for 30 s; then, porcelain beads were added, and the mixtures were treated with a 45-Hz grinding instrument for 10 min and subjected to ultrasonication for 10 min (ice water bath). After standing at −20°C for 1 h, the samples were centrifuged at 12,000 rpm for 15 min at 4°C; the supernatant was carefully removed into an Eppendorf micro test tubes (EP) tube, and the extract was dried in a vacuum concentrator. The extract was added to the dried metabolites (acetonitrile-to-water volume ratio, 1:1), redissolved, vortexed for 30 s, ultrasonicated in an ice water bath for 10 min, and centrifuged at 12,000 rpm for 15 min at 4°C. Ten microliters of each sample was taken for detection in the machine. A hybrid quadrupole-Orbitrap mass spectrometer (Q Exactive; Thermo Scientific, Waltham, MA) was coupled to an ultrahigh-performance liquid chromatography (UHPLC) system (Dionex UltiMate 3000; Thermo Scientific) to perform untargeted metabolomics analysis. An Acquity UPLC HSS T3 1.8um 2.1- by 100-mm column from Waters Corporation (Milford, MA, USA) was used for chromatographic separation with 0.1% formic acid in water (solvent A) and with 0.1% formic acid in ethanol (solvent B). MS1 and MS1-dependent MS2 spectra were acquired at a resolution of *m/z* 37,500. Data were analyzed using Progenesis QI software (Waters), the Human Metabolome Database (HMDB), and the Kyoto Encyclopedia of Genes and Genomes (KEGG) database for metabolite identification; at the same time, the theoretical debris identification was carried out, and the mass number deviation was within 100 ppm.

### Measurement of bacterial DNA by real-time PCR.

Mouse feces DNA was isolated using a stool DNA kit (D4015; Omega Bio-tek, Norcross, GA) following the manufacturer’s instructions. 16S rRNA gene PCRs were monitored on a OneStep Plus instrument (Applied Biosystems, USA), and real-time qPCR was performed with SYBR green master mix (Q141-02; Vazyme Biotech Co., Ltd., Naijing China) according to the manufacturer’s directions. Primers specific to 16S rRNA were used as an endogenous control to normalize loading between samples. The relative amount of 16S rRNA genes in each sample was estimated using the ΔΔ*C_T_* method. Primer sequences were obtained from the Primer Bank primer pairs listed in Table 2.

**TABLE 2:** Bacterial 16S rRNA gene real-time PCR primers

Primer name	Sequence
Bacteroidetes16s F	5′-GAGAGGAAGGTCCCCCAC-3′
Bacteroidetes16s R	5′-CGCTACTTGGCTGGTTCAG-3′
Lactobacillus16s F	5′-AGCAGTAGGGAATCTTCCA-3′
Lactobacillus16s R	5′-CACCGCTACACATGGAG-3′
Firmicutes16s F	5′-GGAGCATGTGGTTTAATTCGAAGCA-3′
Firmicutes16s R	5′-AGCTGACGACAACCATGCAC-3′
A. muciniphila16s F	5′-CAGCACGTGAAGGTGGGGAC-3′
A. muciniphila16s R	5′-CCTTGCGGTTGGCTTCAGAT-3′
Univ Bacterial 16S F	5′-ACTCCTACGGGAGGCAGCAG-3′
Univ Bacterial 16S R	5′-ATTACCGCGGCTGCTGG-3′

### *A. muciniphila* growth conditions.

The strain used in this study was *A. muciniphila* ATCC BAA-835, purchased from Testtop Biotechnology Co., Ltd., Ningbo, China. A. muciniphila was inoculated on brain heart infusion (BHI) agar plates supplemented with 0.5% porcine viscose and 0.05% cysteine. After 48 h culture in 37°C anaerobic jars (Sheldon Manufacturing, USA), the cultured A. muciniphila plate was removed, and single colonies were selected by a one-time inoculation ring method and inoculated into brain heart extract medium. Then, the culture was continued for 48 h in 37°C anaerobic incubator. The cultures were centrifuged (8,000 × *g*, 4°C, 10 min) and washed twice with sterile PBS (pH 7.2). The optical density of PBS solution at 600 nm was measured, and an *A. muciniphila* final suspension with 1 × 10^8^ CFU/mL was obtained. *A. muciniphila* suspended in 200 μL anaerobic PBS (1.0 × 10^8^ CFU per mouse) was orally administered to mice over 14 days.

### Immunofluorescence staining.

Briefly, all sections (5 μm) were routinely deparaffinized in xylene, and antigen was retrieved by means of a 0.01 M sodium citrate-hydrochloric acid buffer. Slides were washed, blocked in 5% normal goat serum for 30 min, and stained overnight at 4°C using primary antibodies, including mouse anti-LC3B antibody (1:2,000; sc-271625; Santa Cruz, Texas, USA) and rabbit anti-NLRP6 antibody (1:1,000; 144-61128-50; Raybiotech, Guangzhou, China), and secondary antibodies conjugated to goat anti-mouse Alexa Fluor 488 (1:400; ab150113; Abcam, Fremont, CA, USA) or goat anti-rabbit Alexa Fluor 594 (1:300; ab150080; Abcam, Fremont, CA, USA). The nucleus was stained by DAPI (4′,6-diamidino-2-phenylindole; C0065, Solarbio, Beijing, China) and washed three times with PBS. The sections were photographed with a Nikon Eclipse TE 2000S inverted microscope (Nikon Instruments, Inc., Melville, NY). The positively stained puncta were counted using Image-Pro Plus software (Media Cybernetics, USA).

### Luminex liquid suspension chip detection to test the cytokines.

Luminex liquid suspension chip detection was carried out by Wayen Biotechnologies (Shanghai, China). The Bio Plex Pro human cytokine group I 23-plex panel was used in accordance with the manufacturer’s instructions. The tissue lysate sample was centrifuged to remove the supernatant, and the protein concentration was determined by the BCA method. In brief, the tissue lysate samples and serum samples were incubated in 96-well plates with microbeads embedded for 1 h and then incubated with detection antibody for 30 min. Subsequently, the values were read using the Bio-Plex MAGPIX system (Bio-Rad).

### Cell treatment.

HT-29 (FS-0269, ATCC) cells were cultured in the Dulbecco’s modified Eagle’s medium (DMEM) (Vivacell) supplemented with 10% fetal bovine serum (FBS) and penicillin-streptomycin (100 ng/mL) at 37°C in a humidified atmosphere with 5% carbon dioxide. The cells were cultured in 96-well culture plates (5 × 10^5^ cells/mL) and 12-well culture plates (5 × 10^4^ cells/mL). Cells were allowed to attach overnight and were then washed twice with PBS and subsequently treated for 24 h in serum-free medium supplemented with different concentration gradients of taurine (HY-B0351; MCE, New Jersey, USA) and histamine (HY-B1204; MCE, New Jersey, USA). Taurine and histamine were dissolved to 1 mM with sterile water and then diluted with medium in a concentration gradient. Finally, the cells were lysed with the lysate, and the total protein was extracted for Western blot analysis.

### HT-29 cells immunofluorescence staining.

Immunofluorescence was used for detecting MUC2 in HT-29 cells. Cells cultured in 48-well plates were fixed with 4% paraformaldehyde for 30 min. Then, membranes were broken with 0.1% Triton X-100 for 10 min and incubated with 5% bovine serum albumin (BSA) at 37°C for 30 min. Rabbit anti-MUC2 (1:1,000; ab272692; Abcam, Fremont, CA, USA) was added to 48-well cell culture plates, which were incubated overnight at 4°C. Subsequently, HT-29 cells were washed on a decolorization shaker with PBS and then incubated with goat anti-rabbit Alexa Fluor 594 (1:300; ab150080; Abcam, Fremont, CA, USA) at room temperature for 60 min. The nucleus was stained with DAPI (C0065; Solarbio, China) and washed three times with PBS. Cells were observed under a fluorescence microscope, and images were collected. The positively stained puncta were counted using ImageJ software (National Institutes of Health, Bethesda, MD, USA).

### Western blotting.

Frozen colon samples were homogenized with radioimmunoprecipitation assay (RIPA) lysis buffer (P0013B; Beyotime Biotechnology, Shanghai, China) to extract total proteins. The homogenate was centrifuged at 12,000 × *g* at 4°C for 20 min. Protein content was quantified using a BCA protein detection kit (CW0014S; CoWin Biotech Co., Inc., Beijing, China) according to the manufacturer’s instructions. These proteins were separated by 10% to 15% SDS-PAGE and then electrotransferred to a polyvinylidene fluoride membrane (Millipore, Billerica, MA, USA). The membrane was then blocked with 5% skim milk at room temperature for 1.5 h. Subsequently, membranes were incubated overnight at 4°C with rabbit antibodies against Bcl-2 antibody (1:1,000; 12789-1-AP; Proteintech, Wuhan, China), Bax antibody (1:5,000; 50599-2-Ig; Proteintech, Wuhan, China), cleaved-caspase-3 antibody (1:1,000; 9661; Santa Cruz, Texas, USA), cleaved-PARP antibody (1:1,000; CSB-PA000080; Huamei biota, Wuhan, China), ATG7 (1:1,000; CSB-PA002294LA01HU; Huamei biota, Wuhan, China), LC3 (1:1,000; NB100-2220, Novus Biologicals, Colorado, USA), ATG5 to ATG12 (1:1,000; A0731; Sigma-Aldrich, USA), claudin-3 antibody (1:1,000; ab15102; Abcam, Fremont, CA, USA), claudin-1 antibody (1:3,000; ab15098; Abcam, Fremont, CA, USA), occludin antibody (1:1,000; ab216327; Abcam, Fremont, CA, USA), phospho-IκB (1:1,000; ab133462; Abcam, Fremont, CA, USA), phospho-NF-κB p65 (1:1,000; ab76302; Abcam, Fremont, CA, USA), caspase-1 (1:1,000; 22915-1-AP; Proteintech, Wuhan, China), ASC/TMS1 (1:1,000; 67824S; Cell Signaling Technology, Massachusetts, USA), NLRP6 (1:1,000; 144-61128-50; Raybiotech, Guangzhou, China), IL-18 (1:1,000; bs-0529R; Bioss, Beijing, China), mouse antibodies against SQSTM1/p62 antibody (1:1,000; 88588; Santa Cruz, Texas, USA), and anti-β-actin antibody (1:10,000; 66009-1-lg; Proteintech, Wuhan, China). Then, membranes were incubated with horseradish peroxidase (HRP)-conjugated goat anti-mouse IgG (1:10,000; SA00001-1; Proteintech, Wuhan, China) (for β-actin) or goat anti-rabbit IgG (1:10,000; SA00001-2; Proteintech, Wuhan, China) for 2 h at room temperature. Immunoblots were performed using an ECL Western blot kit (CW0049; CoWin Biotech Co., Inc., Beijing, China). The bands on the blots were scanned and analyzed with ImageJ (National Institutes of Health, Bethesda, MD, USA).

### Histological analysis.

The intestines were washed with PBS, fixed with 4% paraformaldehyde, and embedded in paraffin. Intestinal tissue sections were stained with hematoxylin and eosin (H&E), and histological scoring was performed in a blind fashion by a pathologist. Intestinal histopathology scores were the combined scores for inflammatory cell infiltration (0 to 4 points), ulcers (0 to 4 points), hyperplasia (0 to 4 points), and crypt distortion area (0 to 4 points) (28). Tissues were stained with AB-PAS. Goblet cells were enumerated in a 100-μm stretch, and at least 30 random fields in six sections of each sample subjected to AB-PAS staining were photographed at a magnification of ×400 with a microscope (BX51; Olympus). Immunohistochemistry was used to stain for proliferating-cell nuclear antigen (Ki-67) and MUC2 in paraffin-embedded intestinal sections. Intestinal tissue sections were incubated with monoclonal rabbit anti-mouse primary antibodies against Ki-67 (1:2,000; ab15580; Abcam, Fremont, CA, USA) and MUC2 (1:2,000; ab272692; Abcam, Fremont, CA, USA) overnight at 4°C. Afterward, the sections were washed with PBS (pH 7.4) and incubated sequentially with biotinylated goat anti-rabbit IgG secondary antibodies (1:300; A0277; Beyotime Co., Ltd., China) for 2 h. Next, following washing, sections were incubated sequentially with 1:300 HRP-streptavidin (1:300; A0303; Beyotime Co., Ltd., China) for 2 h. After treatment with a diaminobenzidine (DAB) kit (CW0125; CoWin Biotech Co., Inc., Beijing, China), an immune response was observed, and hematoxylin was used to counterstain the nucleus for 5 min. Control sections incubated without the primary antibody were examined in all analyses for comparison. For each sample, positive cells were counted in 25 random fields of 5 intestinal cross sections. The average IOD of the positive cells was measured with ImageJ (National Institutes of Health, Bethesda, MD, USA).

### TUNEL staining assay.

The intestines were washed with PBS, fixed with 4% paraformaldehyde, and embedded in paraffin. The tissue was then sectioned (5 μm), and the sections were dewaxed to water. The formalin-fixed colon sections were subjected to TUNEL staining, using a TUNEL assay kit (T2190; Solarbio, China). Nuclei were stained, and images were acquired using a fluorescence microscope (Nikon Instruments, Inc., Melville, New York). The positively stained puncta were counted using ImageJ software (National Institutes of Health, Bethesda, MD, USA).

### Statistical analysis.

Data are expressed as means and standard errors. Data analysis was performed with GraphPad Prism 8.0 (GraphPad Software, San Diego, CA). Two-way analysis of variance (ANOVA) with Tukey’s *post hoc* test for differences between groups was used for statistical analysis and multiple comparisons. A *P* value of <0.05 was considered statistically significant.

### Data availability.

All 16S rRNA gene sequencing read data have been deposited in the NCBI Sequence Read Archive (SRA) repository under accession number PRJNA827692. The remaining data are in the supplemental materials.
