# Supplementary material for: Disruption of the Intestinal Mucosal Barrier Induced by High Fructose and Restraint Stress Is Regulated by the Intestinal Microbiota and Microbiota Metabolites
Source: Microbiol Spectr. 2023 Jan 31;11(2):e04698-22. doi: 10.1128/spectrum.04698-22 (PMC10100858; doi:10.1128/spectrum.04698-22)
Supplement: Supplemental file 1 — Supplemental material. Download spectrum.04698-22-s0001.pdf, PDF file, 0.4 MB [file spectrum.04698-22-s0001.pdf]

Supplementary material method

Quantitative real-time polymerase chain reaction

Total RNA was extracted with TRIzol reagent (CW0580, CoWin Biotech Co., Inc., Beijing, China) according to the manufacturer's protocol. The concentration and purity of RNA was measured with a NanoPhotometer (P330, Implen, Germany). Then, cDNA was synthesized with HiScript QRTsupermix for qPCR (+gDNA wiper) (R312-02; Vazyme Biotech Co., Ltd, Naijing China). Real-time quantitative PCR (qPCR) was performed with SYBR green master mix (Q141-02; Vazyme Biotech Co., Ltd, Naijing China). Changes in fluorescence were monitored on a OneStep Plus instrument (Applied Biosystems, USA). The primers used are shown in Tablel 1.

Table 1: Primers for real-time PCR

Gene name	Forword sequences (5'-3')	Reverse sequences (5'-3')	Source
Muc2	AGGGCTCGGAACTCCAGAAA	CCAGGGAATCGGTAGACATCG	NM_023566.4
Tff3	TTGCTGGGTCCTCTGGGATAG	TACACTGCTCCGATGTGACAG	NM_011575.2
Klf3	GAAGCCCAACAAATATGGGGT	GGACGGGAACTTCAGAGAGG	XM_006503751.5
Lgr5	CAGCCTCAAAGTGCTTATGCT	GTGGCACGTAACTGATGTGG	XM_021173502.2
Olfm4	CAGCCACTTTCCAATTTCACTG	GCTGGACATACTCCTTCACCTTA	XM_021204082.2
Itln1	TGACAATGGTCCAGCATTACC	ACGGGGTTACCTTCTGGGA	XM_029475723.1
Retnlb	AAGCCTACACTGTGTTTCCTTTT	GCTTCCTTGATCCTTTGATCCAC	XM_021185515.1
Ang4	GGTTGTGATTCCTCCAACTCTG	CTGAAGTTTTCTCCATAAGGGCT	XM_021154346.1
Atg5	TGTGCTTCGAGATGTGTGGTT	ACCAACGTCAAATAGCTGACTC	NM_053069.6
Atg7	TGACCTTCGCGGACCTAAAGA	CCCGGATTAGAGGGATGCTC	NM_028835.5
Atg12	GAAGGCTGTAGGAGACACTCCT	GGAAGGGGCAAAGGACTGATTC	NM_026217.3
Beclin1	ATGGAGGGGTCTAAGGCGTC	TCCTCTCCTGAGTTAGCCTCT	NM_019584.4
P62	GAACTCGCTATAAGTGCAGTGT	AGAGAAGCTATCAGAGAGGTGG	NM_011018.3
Nlrp6	TGACCAGAGCTTCCAGGAGT	TTTAGCAGGCCAAAGAGGAA	XM_006536149.4
ASC	ACAGAAGTGGACGGAGTGCT	CTCCAGGTCCATCACCAAGT	NM_023258.4
Caspase-1	CACAGCTCTGGAGATGGTGA	CTTTCAAGCTTGGGCACTTC	XM_021172833.1
IL-18	GACAGCCTGTGTTCGAGGAT	TGGATCCATTTCCTCAAAGG	XM_036154619.1

Measurements of antioxidant activity and lipid peroxidation

The intestinal segments was weighed and placed in a 0.9% saline solution (1:9) to

prepare a homogenate. Finally, the supernatant was extracted by centrifugation (2000 g, 20 min). The total protein concentration was measured using a BCA Protein Assay Kit (CW0014S, CoWin Biotech Co., Inc., Beijing, China). To assess antioxidant capacity, the activities of superoxide dismutase (T-SOD) (A001-1), catalase (CAT) (A007-1), glutathione peroxidase (GSH-Px) (A005-1) activities, as well as total antioxidant capability (T-AOC) (A015-2), and malondialdehyde (MDA) (A003-1) levels were quantified using colorimetric methods from Nanjing Jiancheng Bioengineering Institute (Nanjing, China). Except for MDA, which is expressed in μmol/mg protein, the remaining values are expressed in U/mg protein. These results are detected at specific wavelengths (SOD: 550 nm, CAT: 405 nm, GSH-Px: 412 nm, T-AOC: 520 nm, and MDA: 532 nm).

Microbial DNA extraction and full-length 16S rRNA gene sequencing

The bacterial genomic DNA from frozen colon contents according to the manufacturer's instructions by using a PowerSoil® DNA Isolation kit (MoBio, Shanghai, China). The purity and concentration of the obtained DNA was determined by means of a Nanodrop 1000 (Thermo Fisher Scientific, Wilmington, DE, USA). The preparation of the amplified library uses polymerase chain reaction (PCR) to amplify the full length of 16S rRNA. All amplicon libraries were sequenced using a PacBio SMRT platform (Pacific Biosciences, Menlo Park, United States). The bioinformatics analysis of this study was performed with the aid of the BMK Cloud (Biomarker Technologies Co., Ltd., Beijing, China).

The UCHIME algorithm[24] (V4.2) was used to detect and remove the chimeric

sequence to obtain clean reads. Use USEARCH (V10.0) to cluster sequences with a similarity of 97% into the same operational taxon (OTU), the OTUs with reabundace <0.005% were filtered.[25] The abundance information of OTUs is normalized with the serial number standard corresponding to the sample with the least sequence, and the Alpha diversity and Beta diversity are further analyzed based on the normalized output data. QIIME (V1.8.0), Mothur (V1.3.0) and R software (V3.1) were used for Alpha diversity analysis, including sparseness and Shannon curve, Shannon and Simpson calculations. Beta diversity was calculated by QIIME (V1.8.0) using weighted and unweighted UniFrac distance matrix, including principal coordinate analysis and nonmetric multidimensional scaling heat maps.[26] LDA effect size (LEfSe) analysis was performed to find biomarkers with statistical differences between different groups.[27] Simply put, through LEfSe analysis, LDA threshold>4, using non-parametric factor Kruskal Wallis (KW) and rank test, and then using (unpaired) Wilcoxon rank sum test to identify the most diverse taxa.

Metabolomic analysis

Fecal samples were added to the extract (volume ratio of methanolic acetonitrile to water =2:2:1, internal standard concentration 2 mg/L), vortexed and mixed for 30s, then porcelain beads were added, treated with 45Hz grinding instrument for 10min, and ultrasonic for 10min (ice water bath). After standing at -20 °C for one hour, centrifuge the sample at 12000rpm for 15min at 4°C, carefully remove the supernatant into EP tube, dry the extract in vacuum concentrator, and add the extract to the dried metabolites (acetonitrile to water volume ratio: 1:1) redissolved, vortexed for 30 seconds,

ultrasonicated in an ice water bath for 10 minutes, and centrifuged the sample at 12000rpm for 15min at 4°C, 10μL of each sample was taken for detection on the machine. A hybrid quadrupole-Orbitrap mass spectrometer (Q Exactive, Thermo Scientific, Waltham, MA) was coupled to a UHPLC system (Dionex UltiMate 3000, Thermo Scientific) performed untargeted metabolomics analysis. Acquity UPLC HSS T3 1.8um 2.1×100mm column from Waters Corporation (Milford, MA, USA) was used for chromatographic separation with 0.1% formic acid in water (Solvent A) and with 0.1% formic acid in ethanol (Solvent B). MS1 and MS1-dependent MS2 spectra were acquired at m/z resolution of 37500. Data were analyzed using Progenesis QI software (Waters), the Human Metabolome Database (HMDB), and the Kyoto Encyclopedia of Genes and Genomes (KEGG) database for metabolite identification, at the same time, the theoretical debris identification is carried out, and the mass number deviation is within 100ppm.

Measurement of bacterial DNA by real-time PCR

Mice feces sample DNA was isolated using the stool DNA Kit (Omega Bio-tek, D4015, Norcross, GA) following the manufacturer s instruction. 16S ribosomal DNA PCR reactions was monitored on a OneStep Plus instrument (Applied Biosystems, USA) and Real-time quantitative PCR (qPCR) was performed with SYBR green master mix (Q141-02; Vazyme Biotech Co., Ltd, Naijing China) according to the manufacturer's directions. Primers specific to 16S ribosomal RNA were used as an endogenous control to normalize loading between samples. The relative amount of 16S ribosomal DNA in each sample was estimated using the △△CT. Primer sequences were obtained from

the Primer Bank primer pairs listed in Tablel 2.

Table 2: Bacterial 16S rDNA Real-time PCR primers

**Primer name**	**Sequence**
Bacteroidetes16s F	5′- GAGAGGAAGGTCCCCCAC -3′
Bacteroidetes16s R	5′- CGCTACTTGGCTGGTTCAG -3′
Lactobacillus16s F	5′- AGCAGTAGGGAATCTTCCA-3′
Lactobacillus16s R	5′- CACCGCTACACATGGAG-3′
Firmicutes16s F	5′-GGAGCATGTGGTTTAATTCGAAGCA-3′
Firmicutes16s R	5′-AGCTGACGACAACCATGCAC-3′
A. muciniphila16s F	5′-CAGCACGTGAAGGTGGGGAC -3′
A. muciniphila16s R	5′-CCTTGCGGTTGGCTTCAGAT -3′
Univ Bacterial 16S F	5’-ACTCCTACGGGAGGCAGCAG-3’
Univ Bacterial 16S R	5’-ATTACCGCGGCTGCTGG-3’

A. muciniphila **growth conditions**

The strain used in this study was: *A. muciniphila*. ATCC BAA-835, purchased from Testtop Biotechnology Co., LTD, Ningbo, China. Lines were drawn on brain–heart infusion BHI supplemented with 0.5% porcine viscose and 0.05% cysteine. After 48 h culture in 37°C anaerobic jar (Sheldon Manufacturing, USA), the cultured *A. muciniphila* plate was taken, and single colonies were selected by one-time inoculation ring method and inoculated into brain heart extract medium. Then the culture was continued for 48 h in 37°C anaerobic incubator. The cultures were centrifuged (8000 g, 4C°, 10 min) and washed twice with sterile phosphate buffered saline (PBS, pH 7.2). By measuring the optical density of PBS solution at 600 nm, and *A. muciniphila* final suspension with 1×10^8 colony forming unit (CFU)/ mL was obtained. *A. muciniphila* suspended in 200 µL anaerobic PBS (1.0×10^8 colony-forming units per mouse) was orally administered to mice lasted for 14 days.

Immunofluorescence staining

Briefly all sections (5 μm) were routinely deparaffinized in xylene, antigen retrieval by means of a 0.01M sodium citrate-hydrochloric acid buffer. Slides were washed, blocked in 5% normal goat serum for 30 min, and stained using the primary antibodies overnight at 4 ℃, mouse anti- LC3B antibody (1:2000, sc-271625, Santa, Cruz), rabbit anti-NLRP6 antibody (1:1000, 144-61128-50, Raybiotech, China) and secondary antibodies conjugated to Goat Anti-Mouse Alexa fluor 488 (1:400, ab150113, Abcam) or Goat Anti-Rabbit Alexa fluor 594 (1:300, ab150080, Abcam). The sections were photographed with a Nikon Eclipse TE 2000S inverted microscope (Nikon Instruments,Inc, Melville, New York). The numbers of positively-stained puncta were counted using Image-Pro Plus software (Media Cybernetics, USA).

Luminex liquid suspension chip detection to test the cytokines

Luminex liquid suspension chip detection is completed by Wayen Biotechnologies (Shanghai, China). The Bio Plex Pro Human Cytokine Grp I Panel 23 plex was used in accordance with the manufacturer's instructions. The tissue lysate sample was centrifuged to take the supernatant, and the protein concentration was determined by the BCA method. In brief, the tissue lysate samples and serum samples was incubated in 96-well plates embedded with microbeads for 1 h, and then incubated with detection antibody for 30 min. Subsequently, the values were read using the Bio- Plex MAGPIX System (Bio-Rad).

Cell treatment

HT-29 (FS-0269, ATCC) cells were cultured in the Dulbecco's modified Eagle's medium (DMEM) (Vivacell) supplemented with 10% fetal bovine serum (FBS) and

penicillin/streptomycin (100 ng/ml) at 37°C in a humidified atmosphere with 5% carbon dioxide. The cells were cultured in 96-well culture plates (5 × 10^5 cells/mL) and 12-well culture plates (5 × 10^4 cells/mL). Cells were allowed to attach for overnight, which were then washed twice with PBS and subsequently treated for 24 h in serum-free medium supplemented with different concentration gradients of taurine (HY-B0351, MCE, New Jersey, USA) and histamine (HY-B1204, MCE, New Jersey, USA). Taurine and histamine were dissolved into 1mM with sterile water respectively, and then diluted with medium in concentration gradient. Finally, the cells were lysed with the lysate and the total protein was extracted for Western blot analysis.

HT-29 cells immunofluorescence staining

Immunofluorescence was used for detecting MUC2 in HT-29 cells. Cells cultured in 48 well plates were fixed with 4% paraformaldehyde for 30 min. Then, membrane was broken with 0.1% tritonX-100 for 10 min and incubated with 5% bovine serum albumin (BSA) at 37°C for 30 min. The 48-well cell culture plates were added with rabbit anti-MUC2 (1:1000, ab272692, Abcam, Cambridge, CA, USA) and incubated overnight at 4°C. Subsequently, HT-29 cells were washed on a decolourisation shaker with PBS and then incubated with the goat anti-rabbit Alexa fluor 594 (1:300, ab150080, Abcam, Cambridge, CA, USA), at room temperature for 60min. The nucleus was stained by DAPI (C0065, Solarbio, China) and washed three times with PBS. Cells were observed under a fluorescence microscope and images were collected.

Supplementary Figure 1

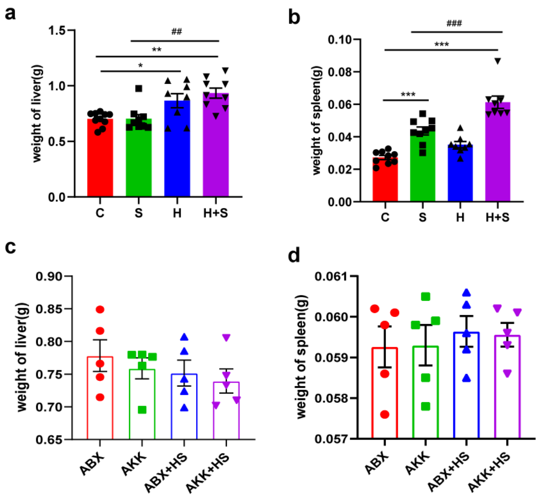

Figure S1. Changes in liver and spleen weight

Changes in liver **(a)** and spleen **(b)** weights induced by high fructose feeding and restraint stress (n=8-10). Changes in liver **(c)** and spleen **(d)** weight were caused by antibiotic treatment and colonization of *A. muciniphila* (n=5). Data are presented as the mean ± SEM. *P < 0.05; **P < 0.01; ***P < 0.001 indicate significant difference. #P < 0.05; ##P < 0.01; ###P < 0.001 compared with the restraint stress group (S) indicate significant difference. C: control group; S: restraint stress; H: high-fructose; H+S: high-fructose and restraint stress. ABX: Antibiotic treatment group; AKK: *A. muciniphila* colonization group; ABX+HS: Antibiotic treatment + high fructose feeding and restraint stress stimulation group, AKK+HS: *A. muciniphila* colonization + high fructose feeding and restraint stress stimulation group.

Supplementary Figure 2

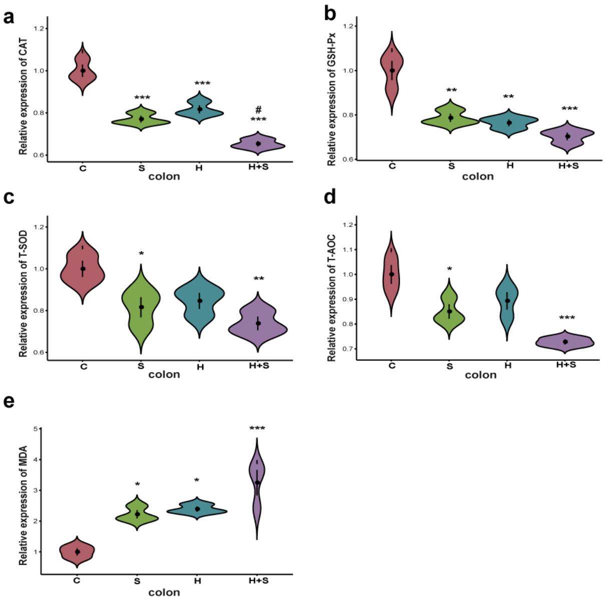

Figure S2. Effects of restraint stress and high fructose on oxidative stress in the colon of mice.

These graphs show the contents of catalase (CAT) **(a)**, glutathione peroxidase (GSH-Px) **(b)**, total superoxide dismutase (T-SOD) **(c)**, total antioxidant capacity (T-AOC) **(d)**, and malondialdehyde (MDA) **(e)** in the colon of the control, stress, high fructose and high fructose+ stress. The results represent the mean ± SEM for each group (n=5 animals). Data are presented as the mean ± SEM. *P < 0.05; **P < 0.01; ***P < 0.001indicate significant difference. #P < 0.05; ##P < 0.01; ###P < 0.001 compared with the restraint stress group (S) indicate significant difference. C: control group; S: restraint stress; H: high-fructose; H+S: restraint stress and high-fructose.

Supplementary Figure 3

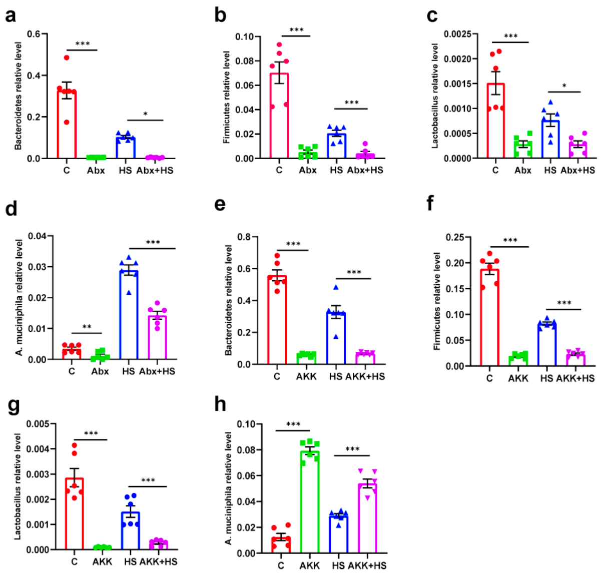

Figure S3. Changes in fecal flora abundance after antibiotic treatment and *A. muciniphila* colonization

Relative abundance of *Bacteroides* **(a)**, *Firmicutes* **(b)**, *Lactobacillus* **(c)** and *A. muciniphila* **(d)** in feces after the antibiotic treatment (n=5) were quantitated by 16SrDNA qPCR analysis. Relative abundance of *Bacteroides* **(e)**, *Firmicutes* **(f)**, *Lactobacillus* **(g)** and *A. muciniphila* **(h)** in feces after *A. muciniphila* colonization (n=5) were quantitated by 16SrDNA qPCR analysis. Data are presented as the mean ± SEM. *P < 0.05; **P < 0.01; ***P < 0.001 indicate significant difference. C: control group; S: restraint stress; H: high-fructose; H+S: high-fructose and restraint stress. ABX: Antibiotic treatment group; AKK: *A. muciniphila* colonization group; ABX+HS: Antibiotic treatment + high fructose feeding and restraint stress stimulation group, AKK+HS: *A. muciniphila* colonization + high fructose feeding and restraint stress stimulation group.
